# Multi-scale assessment of roost selection by ‘ōpe‘ape‘a, the Hawaiian hoary bat (*Lasiurus semotus*)

**DOI:** 10.1371/journal.pone.0288280

**Published:** 2023-08-24

**Authors:** Kristina Montoya-Aiona, P. Marcos Gorresen, Karen N. Courtot, Aaron Aguirre, Flor Calderon, Sean Casler, Sarah Ciarrachi, Julia Hoeh, Josephine L. Tupu, Terry Zinn

**Affiliations:** 1 U.S. Geological Survey, Pacific Island Ecosystems Research Center, Kīlauea Field Station, Hawai‘i National Park, Hawaii, United States of America; 2 Hawai‘i Cooperative Studies Unit, University of Hawai‘i at Hilo, Hawai‘i National Park, Hawaii, United States of America; University of Reunion Island, RÉUNION

## Abstract

The Hawaiian hoary bat (*Lasiurus semotus*; Chiroptera: Vespertilionidae), commonly and locally known as ‘ōpe‘ape‘a, is a solitary, insectivorous, and foliage-roosting species distributed across a wide range of habitats in lowland and montane environments. The species, as with many others in the Hawaiian archipelago, are facing a suite of challenges due to habitat loss and degradation, introduced predators and pests, and climate change. An understanding of the roost requirements of foliage-roosting tree bats is critical to their conservation as these habitats provide several important benefits to survival and reproduction. Because little is known about ‘ōpe‘ape‘a roost ecology and considerable effort is needed to capture and track bats to roost locations, we examined resource selection at multiple spatial scales—perch location within a roost tree, roost tree, and forest stand. We used a discrete choice modeling approach to investigate day-roost selection and describe attributes of roost trees including those used as maternity roosts. ‘Ōpe‘ape‘a were found roosting in 19 tree species and in an assortment of landcover types including native and non-native habitats. Our results are largely consistent with findings of other studies of foliage-roosting, insectivorous tree bats where bats selected roost locations that may offer protection and thermoregulatory benefits.

## Introduction

Bat species globally face threats from habitat loss or degradation, disturbance, pathogens and infectious diseases, invasive species, climate change, and wind energy [1, 2]. The Hawaiian hoary bat (*Lasiurus semotus*; Chiroptera: Vespertilionidae) [3–6] commonly and locally known as ‘ōpe‘ape‘a, is a solitary, insectivorous, and foliage-roosting species distributed across a wide range of habitats in both lowland and montane environments [7]. Listed as state and federally endangered [8], it is the only extant, endemic, terrestrial mammal in the Hawaiian archipelago and is culturally significant in Hawai‘i, appearing in the Kumulipo or Hawaiian creation chant [9]. The species, as with many others in the Hawaiian archipelago, is facing a suite of challenges due to habitat loss and degradation, introduced predators and pests, and climate change [10]. Specific threats to the ‘ōpe‘ape‘a include collisions with wind turbines [11], loss of roosting and foraging habitat, roost disturbance, and unknown effects of pesticides and predation [8, 12]. Lack of information about population dynamics, life history, habitat selection, and threats to the species has made it difficult for managers to design effective recovery and mitigation strategies. Several studies have identified forests as an important resource for the species, particularly in relation to foraging habitat [13–15], but none have examined habitats specifically used for roosting.

Like other foliage roosting bats, ‘ōpe‘ape‘a spend much of their time in day-roosts. In addition to resting locations, day-roost habitats can provide several critical functions including shelter from weather and predators, and sites to rear young [16–18]. Data on roost use is needed to help inform forest restoration and mitigation efforts that aim to aid in the recovery of the species. However, it is extremely difficult to identify ‘ōpe‘ape‘a day-roost locations because of their relatively small size and cryptic nature, which limits tracking device options and visibility at roost. As such, no studies have examined roost habitat selection, day-roost trees have only been described anecdotally in general terms of tree species observed or suspected as roosts [19, 20], and only a few maternity roosts have ever been found or described [21].

In this first ever study of ‘ōpe‘ape‘a roost use, we used a discrete choice modeling approach to investigate day-roost selection and describe attributes of roost trees, including those used as maternity roosts. Many studies of bat roost ecology focus on a single scale (e.g., roost tree or forest stand). However, because little is known about ‘ōpe‘ape‘a roost ecology and considerable effort is needed to capture and track bats to roost locations, we examined resource selection at multiple spatial scales—perch location within a roost tree, roost tree, and forest stand. For roost selection models, we used a suite of roost habitat attributes common to foliage-roosting bats (e.g., [22–27]). We hypothesized that several factors, operating at different spatial scales, would influence day-roost selection. At the perch level, we hypothesized bats would select perches oriented toward the southern aspect of the tree and those highest off the ground with greater percent canopy cover. At the roost tree level, we hypothesized that bats would select day roost sites with physical features that distinguish them from randomly available trees such as height, diameter at breast height, and canopy cover. Particularly, we hypothesized that bats would select roosts in trees that were taller, larger in diameter, and have greater canopy cover compared to other trees in the same stand. Moreover, the selection of specific roost sites by reproductive female bats, particularly those with pups, would provide net benefits (e.g., protection from predation, thermoregulation) when compared to randomly available trees and roosts of non-reproductive female and male bats. At the broader habitat forest stand-level, we hypothesized that bat roost selection on the landscape would be driven by proximity to forest edges and forest community composition (i.e., native versus non-native). Identifying factors influencing roost site selection and characteristics of roost-tree habitat could be used to help inform restoration and mitigation efforts aimed at species recovery.

## Methods

### Study area

The study area covered approximately 2,200 km^2^ on the windward or east side of Hawai‘i Island from the Kupapaulua to Ka‘ahakini watersheds, as well as approximately 990 km^2^ in the Keāhole, Honokōhau, and Ki‘ilae watersheds on the leeward or west side of Hawai‘i Island. This area included native and non-native mixed forests, timber plantations, agro-ecosystems, and urban/suburban landscapes from sea level to approx. 2,300 m elevation. These areas were selected because of previously demonstrated high levels of ‘ōpe‘ape‘a presence and/or activity [7, 15].

### Bat capture and tracking

Bats were captured by mist-netting at multiple locations across Hawai‘i Island from May 2018 through August 2021, under U.S. Fish and Wildlife Service (USFWS) permit TE-003483 and State of Hawai‘i permits WL18-18, WL19-19, and WL19-52 and protocols approved by the University of Hawai‘i, Institutional Animal Care and Use Committee (protocol #04–039). Netting locations were selected to include a range of elevations and land-cover types (see [28]). Age, sex, reproductive condition, tissue, and guano samples were obtained from each captured bat whenever possible. Age was classified as either adult or juvenile and assessed based on the degree of epiphyseal-diaphyseal fusion [29]. Unique, individually color-coded split-ring plastic forearm bands (3.1-mm darvic solid color, celluloid striped; Avinet Research Supplies, Portland, Maine, USA) were affixed for identification of individuals if recaptured or resighted at a roost. Before release, very high frequency (VHF) radio-transmitters (Model PIP3, transmitter mass 0.63–0.73 g; Biotrak Ltd., Wareham, UK) were attached with surgical glue (Perma-Type Surgical Cement, Perma-Type Company Inc., Plainville, Connecticut; or Torbot Bonding Cement, Torbot Group, Inc., Cranston, Rhode Island) to the interscapular region on the posterior of the bat only when the transmitter weighed less than 5% of bat mass. Radio-tagged bats were tracked using VHF receivers (model TRX-1000, Wildlife Materials Inc., Murphysboro, Illinois and/or model R410, Advanced Telemetry Systems, Isanti, Minnesota) tuned within the 164.000–164.999 MHz range. Receivers were equipped with omnidirectional (model SN-150, Cushcraft, Starkville, Mississippi; model 500C, Wildlife Materials Inc., Murphysboro, Illinois) or directional three- or five-element collapsible Yagi antennas (models F164-165-3FB and F164-165-5FB, Wildlife Materials Inc., Murphysboro, Illinois). Tracking was conducted on the ground, from vehicles, and by foot during daytime hours in teams of 2–4 personnel. Vehicles were driven along passable routes with an omnidirectional antenna affixed to the roof or a directional antenna extended from a window. When possible, field personnel hiked into forest stands with directional antennas and receivers. Upon detection of a radio signal, global positioning system (GPS) coordinates and compass bearings of the signal direction were recorded. Data were recorded at multiple locations and mapped to narrow down the radio-tagged bat’s location. When a roost tree was confirmed, a GPS coordinate was recorded at its location.

### Roost tree and habitat identification

After using the radio signal to determine if a radio-tagged bat was in a tree or group of trees, various tools were used to locate and confirm the bat at perch within a roost tree. A handheld thermal imager (model Ti450 or Ti480, Fluke Corporation, Everett, Washington) was used to scan possible roost tree(s) to determine a heat signature of a roosting bat. Binoculars and/or spotting scope were used to view a bat within a roost tree. When necessary, field personnel observed the area near dusk to identify the tree and in some cases perch, from which the bat emerged. Opportunistic searching of a subset of known or suspected roost tree areas was conducted, when possible, with emphasis on previously used maternity roosts. Any bats that were observed but not captured and banded during this study were categorized as an “unknown” individual, but roost tree data were collected. Single bats identified by this method were categorized as unknown sex. When multiple bats were observed roosting together and exhibiting maternal behavior [21], such as nursing or grooming during the reproductive season, they were assumed to be a family group (mother with pups) and the tree was designated as a female maternity roost.

In cases where a specific roost tree was not able to be confirmed, only broader habitat level (“stand”) characteristics were obtained from an approximated roost tree location. The estimated coordinates of the bat’s roost location were derived from point location and compass bearings collected during radio-tracking, using Location of a Signal (LOAS) software (version 4.0.3.8, Ecological Software Solutions LLC, Urnäsh, Switzerland). For purposes of habitat classification, each roost tree had an associated roost stand that was defined as a 50-meter radius buffer around the roost tree location point or estimated coordinates when derived from LOAS. All roost trees identified and confirmed had an associated roost stand, but in instances where roost trees were not identified, only stand-level characteristics were examined.

Roost trees were classified to the species level, and roost tree height, diameter at breast height (DBH), percent canopy cover, distance to nearest tree, and elevation were measured. Additionally, if a bat was spotted at its perch within the roost tree, then percent canopy cover, aspect, and height of the bat’s perch were also recorded. Perch locations were considered distinct when they were more than 1 m away from a previous perch location. For the comparison of used roost and random trees, we identified random trees (≥ 10-cm DBH to exclude understory saplings from analysis) at random bearings and distances within a 50-m radius of each roost tree. For each random tree we recorded species, height, and DBH. Tree height and bat perch height (if known) were measured using a laser range finder (Model Forestry Pro Laser Rangefinder/ Hypsometer, Nikon USA, Mellville, New York). Tree DBH was measured by wrapping a diameter tape (Model 283D/10M, Forestry Suppliers, Jackson, Mississippi) around the tree at breast height (1.3 m) from the base of the tree. A tree was measured and classified as a single-bole tree if multiple trunks forked at a point higher than 1.3 m. If the trunks forked at a point lower than 1.3 m, the tree was classified as multi-trunked and DBH was measured for each trunk and summed. Additionally, if the tree was growing on a slope, DBH was measured on the top part of the slope. Percent canopy cover of roost trees was measured using a spherical densiometer (Convex Model A, Forestry Suppliers, Jackson, Mississippi) and averaged using four readings taken around a roost tree. Personnel positioned their back toward the reference tree and moved around the tree facing North, East, South and West. Similarly, the estimated percent canopy cover of the bat perch location was measured using a spherical densiometer with personnel taking four readings directly underneath the bat perch facing North, East, South and West. Roost perch aspect (i.e., compass direction) was measured using a compass (Model M-3, Suunto, Vantaa, Finland).

For each roost stand we collected topographic and forest habitat data. We used ArcGIS 10.6 (Esri 2017) to produce measures of the elevation, slope, and topographic position from a 1/3 arc-second (approx. 10 m) resolution digital elevation model [30]. We classified the location of stands in relation to the surrounding terrain with a topographic position index [31]. Index values for stands were calculated as their elevation relative to the average of surrounding cells within a radius of 50 m and defined as situated within a drainage (valley/gully: ≤-60), lower, mid or upper slope (>-60 to 60), or ridge (rim/peak: >60). Distance from centroid (either roost tree or approximated roost tree location) to forest edge, and mean canopy height in meters were measured with Pictometry software (Version 2-14-8-380, Eagle View Technologies, Bellevue, Washington). Forest edge was defined as the limit of continuous canopy at or near boundary between two adjacent land cover types with ≥50% of tree’s canopy open and not interlocking with adjacent tree’s canopy’s [32]. Mean canopy height was determined by measuring 10 random canopy trees within the defined roost stand using the Pictometry height tool. Habitat classification of tree crown cover, tree height, tree species composition, and understory and ground cover were described using the methodology detailed in Jacobi [33], which is a hierarchical classification system used for describing vegetation communities. The concatenated code describes habitat type in this order: tree canopy cover + height class + overstory tree composition (species association type: species dominance) + habitat species association type + understory species composition (species association type: species dominance) + other information. Habitat classifications were completed using Pictometry software images and included ground-truthing in the field when possible. Over 20 classes were identified, and for the purposes of modeling, these were grouped into four classes based on the relative prevalence of the dominant tree species: *Metrosideros polymorpha* (common name: ‘ōhi‘a lehua (hereafter, ‘ōhi‘a); a widespread endemic forest and woodland tree species), *Melaleuca quinquenervia* (common name: paperbark; a non-native species planted frequently at field borders), *Eucalyptus* species (a broad assortment of non-native trees used in timber tree plantations), and miscellaneous non-native invasive species (e.g., *Schinus terebinthifolius*, *Psidium cattleianum*, *Melochia umbellata*). Canopy tree species were also classed as an additional binary variable indicating a native versus non-native origin. Canopy cover as an attribute of crown density at the tree-level (“foliage opacity”; *sensu* [34]) was classed as closed (≥60%) or open (<60%). Canopy cover as a combined attribute of the canopies and spacing of multiple trees at the stand-level was classed as closed (>60%), open (25–60%), and scattered (<25%).

### Statistical analyses

We characterized day-roost habitat use at three spatial extents—perch, tree, and stand. Perch-level analyses examined patterns of roost perch use related to canopy cover, perch height (relative to tree height), and aspect (i.e., cardinal direction, a circular variable). We used the Rayleigh test of uniformity to determine whether perch aspect for all bats differed from that of a random distribution, and the Watson-Wheeler test to compare the equality of aspect values as a function of sex and between reproductive and non-reproductive periods. We used a two-sided two-sample Kolmogorov-Smirnov (KS) test of sex-based differences in the use of perch canopy cover and relative height (i.e., perch height adjusted for total tree height), and a two-sided one-sample KS test to examine if these attributes differed from random for bats by comparing the observed distribution to a uniform distribution.

We examined roost preferences at the tree- and stand-level with discrete choice analysis. The method compares selected to available but unselected random sites by estimating the probability of specific habitat attributes being used [35]. Discrete choice analysis allows for the concurrent comparison of several habitat types and can incorporate dependence introduced by repeated measurements of individual animals [36]. In contrast with maximum entropy (Maxent) and logistic regression methods, discrete choice modeling is better able to estimate selection distributions regardless of sample size or the number of random locations used in the analyses [37]. The method has been used effectively to examine roost and foraging selection by bats [38–40].

Discrete model choice sets were developed based on distinct selection events and served as the observational units at each level (roost tree and roost stand). The number of choice sets was determined both by the number of unique roost sites to which a bat was tracked and the duration of the sampling period over which it was confirmed at one or more roosts. A “basic” choice set was composed of one used site and two random sites for each selection event. For bats observed for a short period (<3 days) at only one roost, we produced a choice set limited to only a single selection event. For bats tracked to only one roost but confirmed at that roost on at least three days, we included an additional independent selection event for that roost. An additional selection event was also assigned to bats that returned to the same roost locations during more than one season (reproductive season = May to September; non-reproductive season = October to April) and/or year. Bats that used multiple roosts were assigned an equivalent number of selection events, and additional events if confirmed at a particular roost on at least three days. Although it is appropriate to include multiple choice sets for single individuals [35], deriving selection events and their respective choice sets for every day during which an individual was observed at a site, risks biasing selection patterns towards individuals tracked for longer periods of time and/or using multiple sites. Incorporating a restricted number of additional selection events based on observations obtained during multiple observation days was used as a balance between avoiding bias from repeated use of the same site and using information for individual bats demonstrating sustained fidelity to a roost.

To develop a choice set for each individual bat at the scale of the roost stands, we identified two random locations within 2.5 km of the selected location; a threshold derived from the median distance between the capture location and observed roost location(s). Restricting random sites in this manner ensured that the area used to define the choice set was likely to be available to an individual and did not contain resources that were inaccessible due to excessive distance from the used site [37]. The choice set developed at the tree-level was derived from randomly selected trees within 50 m of the roost tree and reflected the available resources of neighboring trees within a stand used for roosting. We tested for collinearity between pairs of predictor variables; those exceeding a correlation coefficient threshold of |0.35| were not included together in the same set of models. Tests were done with Pearson’s correlation for continuous predictor variables, point-biserial correlation for continuous and dichotomous variables, and Cramer’s V for multinomial categorical variables. We also excluded from the input data a single roost in low-stature matted fern (uluhe or false staghorn fern, *Dicranopteris linearis*) as it did not allow for standard measures of tree physiognomy.

The candidate set of predictor variables yielded 48 models for the tree-level and 128 models for the stand-level analyses (including a null model for each level; S1 Appendix). We fit our discrete choice models in R (version 4.1.0; R Core Team 2021) with the package “mlogit” [41]. To compare models within each group we used small-sample-size corrected Akaike information criterion (AICc) via the AICctab function of the R package “bbmle” [42, 43]. We only discuss models with ΔAICc ≤ 4 because those with higher ΔAICc values have considerably less support [42]. Given our uneven sex ratio and small sample size, we did not analyze our tree- and stand-level data for potential sex-specific roost use and selection.

As traditional goodness of fit measures are not appropriate for matched case control designs such as those used in discrete choice analysis [44], we validated our top ranking models using a k-fold approach modified to apply to choice sets. We used a random subset of 80% of the choice sets to train a model and retained the remaining 20% of choice sets to test the predictive capability of the trained model (while maintaining the 1:2 ratio of used to randomly available resources). We repeated the process 10 times and at each iteration, calculating the relative probability of selection for each test set. We assessed a model’s performance by determining the percentage of used roost sites in the testing set that were correctly predicted. A value >33% (the ratio of 1 used roost to 2 random sites in each choice set) indicated the model performed better than would be expected at random (e.g., [38, 45, 46]). Significance was assessed at α = 0.05 for all statistical tests. We report the validation results for each of the top-ranked models in the tree- and stand-level analyses. Predictor variables that were significant in the discrete choice models were further examined individually for potential differences between female and male bats. We present the values from discrete choice models and descriptive statistics as the mean ± standard deviation (SD).

## Results

**Fig 1:**
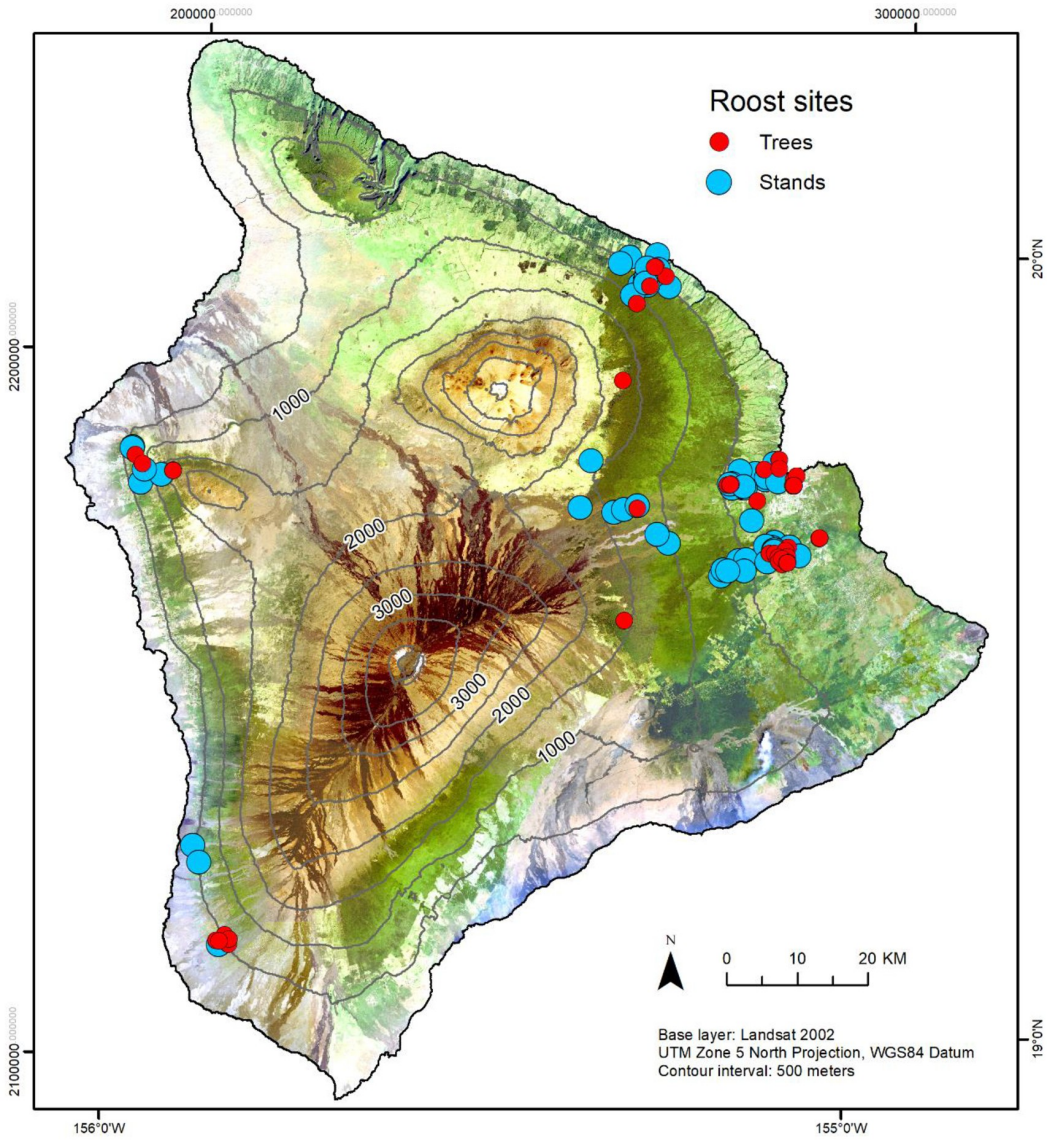
Locations of ‘ōpe‘ape‘a (*Lasiurus semotus*) roost trees and stands identified May 2018–August 2021.

A total of 148 bats (37 female; 101 male) were captured from May 2018 to August 2021, including 10 recaptures. Bats of small body size were not radio-tagged if the transmitter-to-body weight ratio exceeded 5% (see [47, 48]). We attempted to radio track 127 bats to roosting locations (Fig 1). There were 36 bats (6 female; 30 male) that we were unable to track to any roost level after tagging and release. The distance from capture location to roost location ranged from 8 m to 24,626 m (mean 3,618 ± 4,671 m; median 2,513 m).

### Perch-level analyses

We observed a total of 69 perches used by 52 bats (16 female; 22 male; 14 unknown) within 44 roost trees. In some instances, a roost tree was identified but the exact location of the bat within the tree could not be reliably confirmed, therefore perch information was not collected. We identified only one perch location for most roost trees. However, five roost trees had multiple perch locations identified within them over the course of the study (perches per tree: 2, 2, 4, 5, 17). Alternatively, nine bats (2 female; 7 male) used more than one perch among multiple trees (range 2–5; mean 3 ± 1).

**Fig 2:**
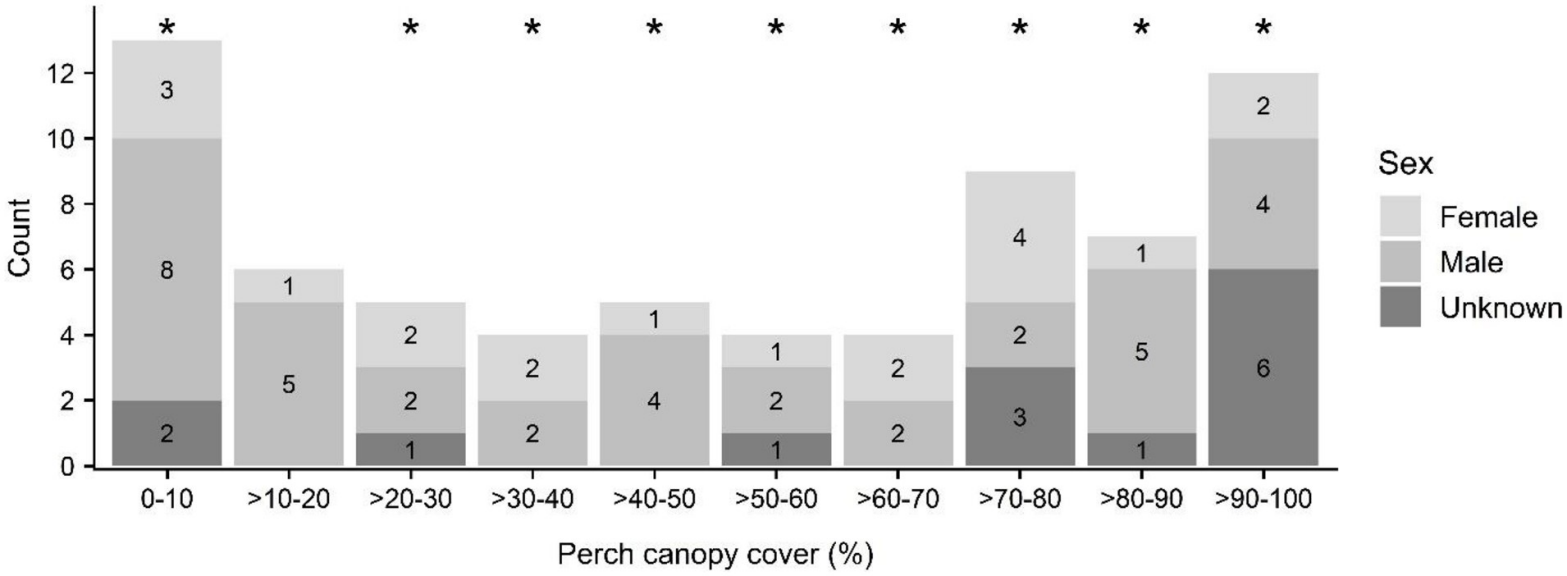
‘Ōpe‘ape‘a (*Lasiurus semotus*) roost perch canopy cover by sex. Cover classes used as maternity roosts are indicated with asterisks.

Perch canopy cover (i.e., foliage transparency directly above perches) was bi-modally distributed, with the majority of observations at either extreme relative to mid-range values, and differed significantly from random for bats as a whole (KS D(69) = 1.0, p-value < 0.001) (Table 1, Fig 2). Cover varied widely for both females and males (range 4%– 99% for both sexes; female: 51 ± 32%; male: 45 ± 34%), and did not differ significantly by sex (KS D(19,36) = 0.21, p-value = 0.683).

**Table 1:** Statistics of predictor variables measured at the perches of roosts used by female (n = 19) and male (n = 36) ‘ōpe‘ape‘a (*Lasiurus semotus*).

	Female				Male			
	Mean	SD	Min	Max	Mean	SD	Min	Max
Perch canopy cover (%)	51	32	4	99	45	34	4	99
Perch height (m)	14	6	5	24	12	5	2	29
Relative perch height (%)	70	21	28	97	71	21	17	97
Aspect (degrees)	221	72	25	334	199	82	48	326

**Fig 3:**
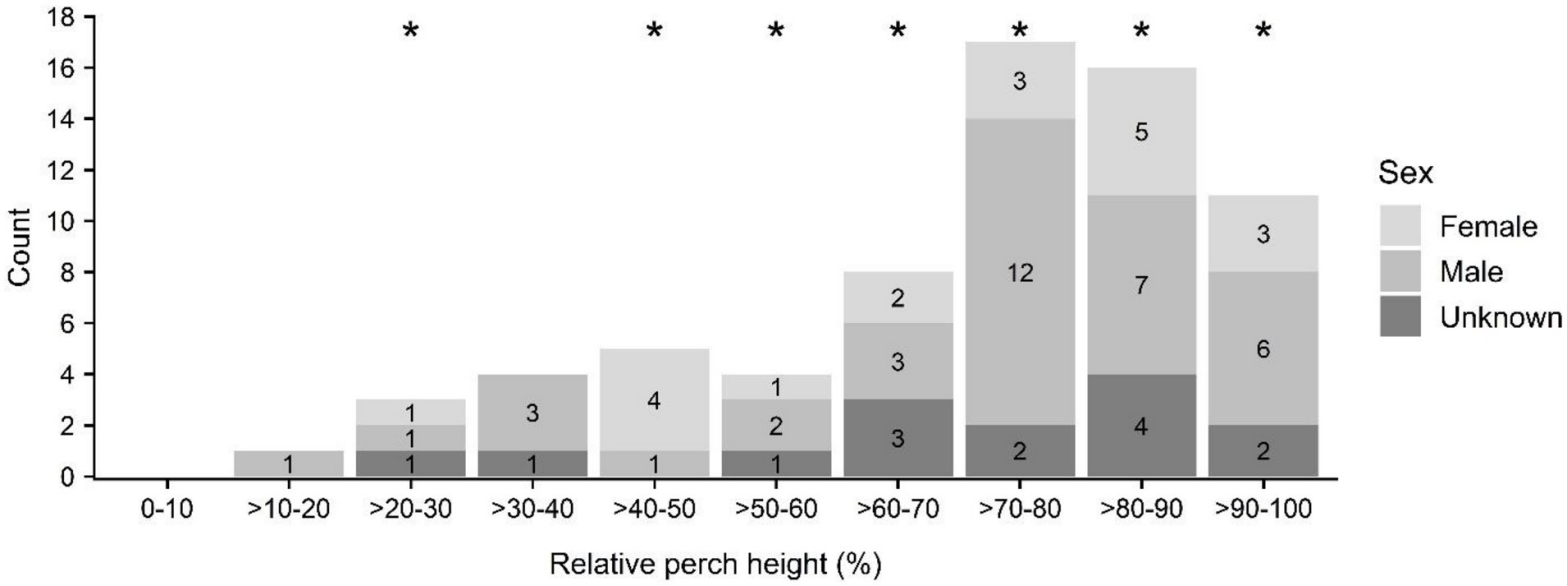
‘Ōpe‘ape‘a (*Lasiurus semotus*) roost perch height (relative to tree height) by sex. Height classes used as maternity roosts are indicated with asterisks.

Bats largely perched in the upper third of trees (relative to total tree height) (female: 70 ± 21%; male: 71 ± 21%), and this behavior differed significantly from random for bats as a whole (KS D(69) = 0.365, p-value < 0.001) (Fig 3). Absolute perch height varied widely (female: 5 to 24 m; mean 14 ± 6 m; male: 2 to 29 m; mean 12 ± 5 m) and did not differ significantly by sex (KS D(19,36) = 0.218, p-value = 0.600). The 2-m perch height recorded for a male bat is noteworthy for it having been a roost in uluhe (*Dicranopteris linearis*), a low-stature matted fern. Relative perch height was negatively, if marginally, correlated with absolute tree height (*r* = -0.23, p-value = 0.061); that is, perches in smaller trees tend to occur in the upper parts of the tree.

**Fig 4:**
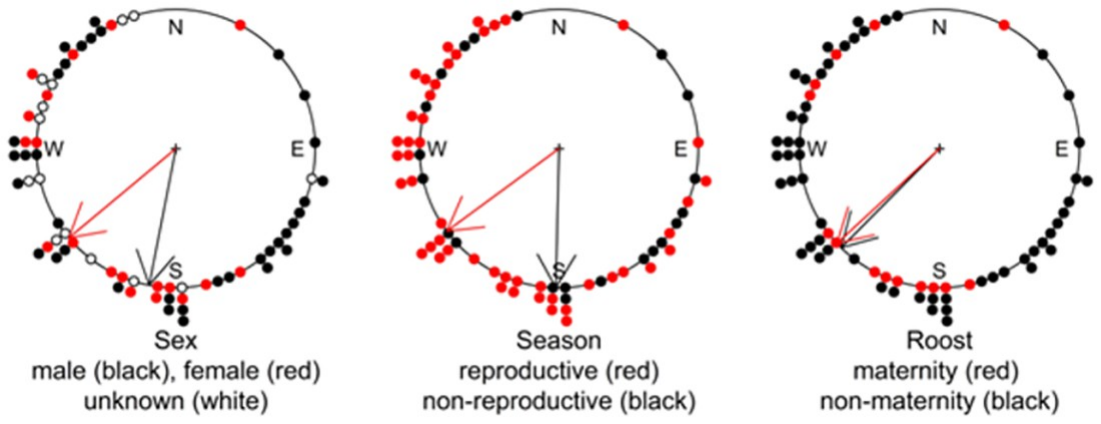
Mean (arrow) aspect of roost perches (points) used by male and female ‘ōpe‘ape‘a (*Lasiurus semotus*), by season and by maternity groups.

The mean aspect of roost perches for all bats was significantly oriented toward the southwest (225°) (Rayleigh test statistic = 0.355, p-value < 0.001). However, mean aspect was not found to significantly differ between female (230°) and male (191°) bats (Watson-Wheeler two-sample test of homogeneity *W* = 4.787, p-value = 0.091), between reproductive (May–Sept) (234°) and non-reproductive (Oct–Apr) (181°) seasons (*W* = 4.727, p-value = 0.094), or between roosts used by individuals or groups at maternity (227°) and non-maternity roosts (224°) (*W* = 2.129, p-value = 0.345) (Fig 4).

### Tree-level analyses

A total of 56 roost trees were used by 46 bats (18 female; 25 male; 3 unknown). Five roost trees were used by multiple bats (range 2–12; mean 4 ± 4) throughout the study period and in some cases concurrently. Alternatively, 11 bats (2 female; 9 male) used more than one roost tree (range 2–6; mean 3 ± 1). During the course of tracking and revisiting known roost trees and surrounding areas, an additional six roost trees were identified with unknown, not previously captured bats. Of these six trees, three were classified as maternity roost trees where an adult female was sighted roosting with pups and displaying maternal behavior.

**Fig 5:**
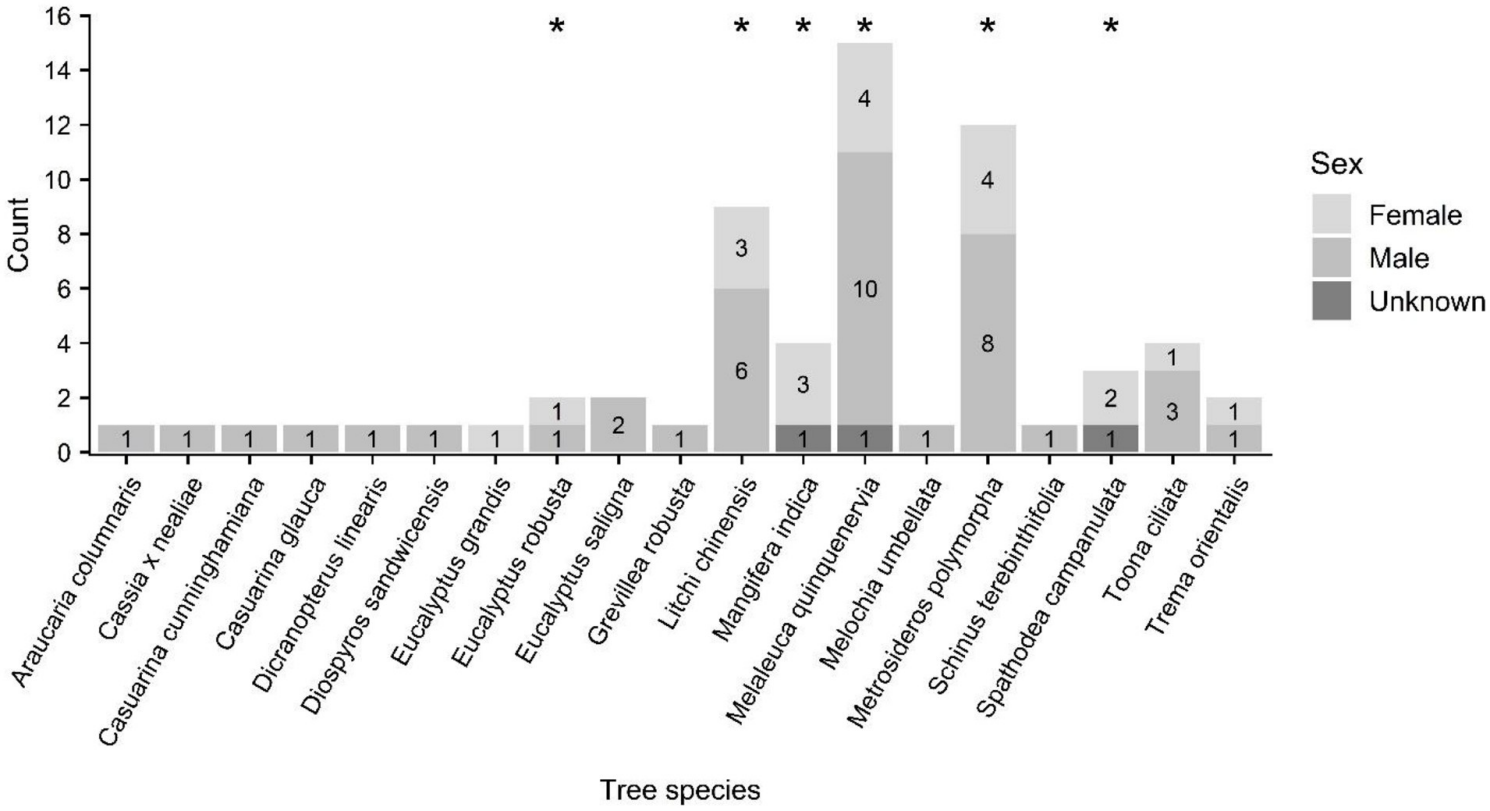
Roost tree species used by ‘ōpe‘ape‘a (*Lasiurus semotus*) by sex. ‘Ōhi‘a (*Metrosideros polymorpha*) and lama (*Diospyros sandwicensis*) are endemic tree species, and uluhe (*Dicranopteris linearis*) is an indigenous matted fern. All other species listed are non-native. Tree species observed used as maternity roosts are indicated with asterisks.

**Fig 6:**
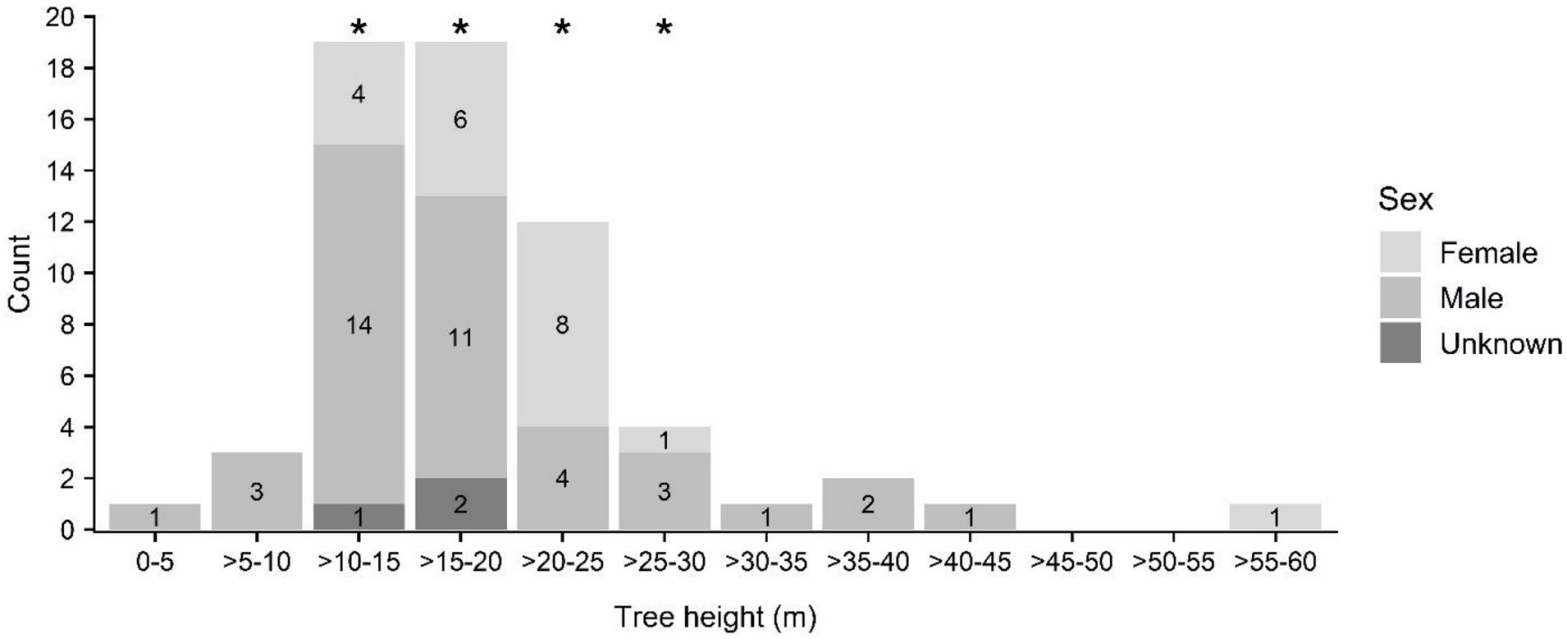
Height classes of roost trees used by ‘ōpe‘ape‘a (*Lasiurus semotus*) by sex. Height classes of trees used as maternity roosts are indicated with asterisks.

**Fig 7:**
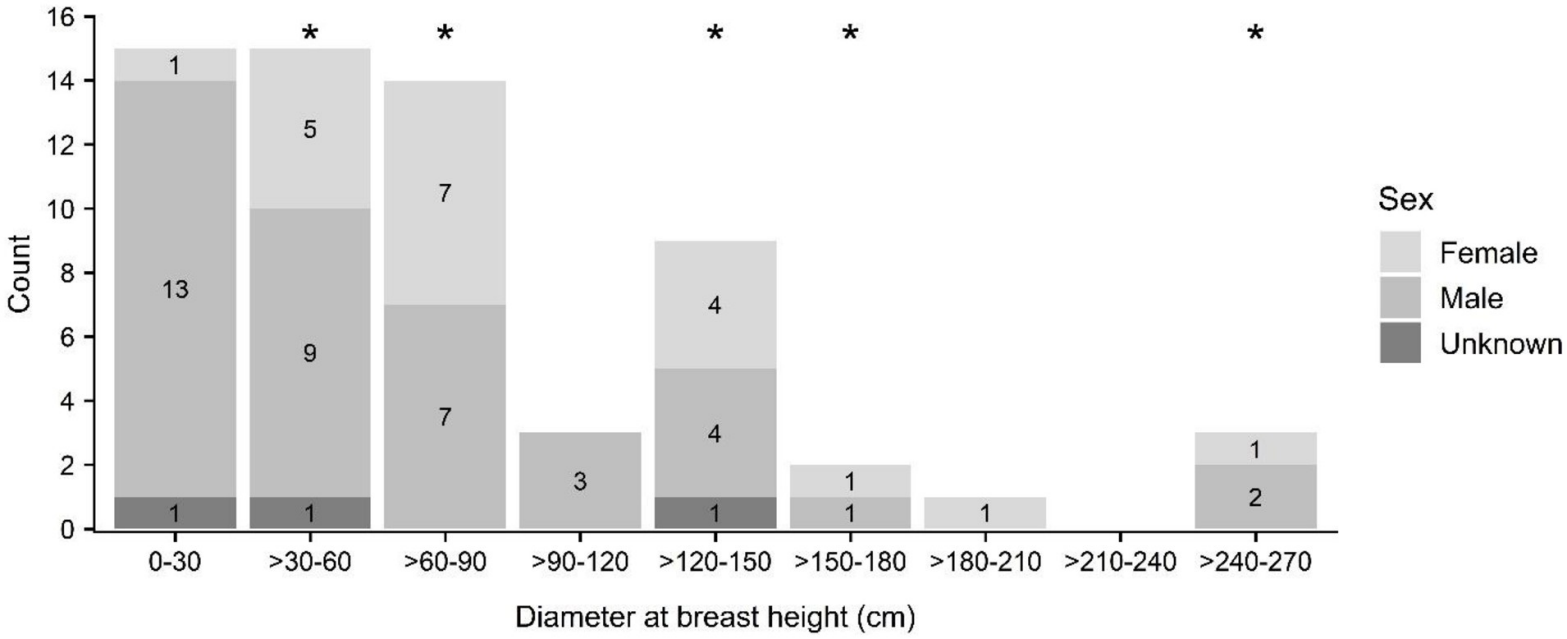
Diameter at breast height (DBH) of roost trees used by ‘ōpe‘ape‘a (*Lasiurus semotus*) by sex. Diameter classes of trees used as maternity roosts are indicated with asterisks. We excluded a single roost in low-stature matted fern (uluhe or false staghorn fern, *Dicranopteris linearis*) as it did not allow for standard measures of tree physiognomy.

Tree species in which roosting bats were located were primarily composed of non-native plantation species (e.g., paperbark (*Melaleuca quinquenervia*); n = 15) and fruit trees in residential settings (e.g., lychee (*Litchi chinensis*), mango (*Mangifera indica*); n = 13), although ‘ōhi‘a (*Metrosideros polymorpha*; n = 12), a native species widespread at mid- and upper elevations, was also prominently represented (Fig 5, see S2 Appendix). Roost tree height ranged from 5 to 56 m (mean 19 ± 8 m) (Fig 6) while DBH ranged from 12 to 268 cm (mean 78 ± 61 cm) (Fig 7). The height of trees used by roosting bats (mean 19 ± 8 m) differed significantly from random (mean 15 ± 10 m) for bats as a whole (KS D(62) = 1.0, p-value < 0.001), but not between female and males (female: mean 21 ± 9 m; male: mean 18 ± 8 m; KS D(20,39) = 0.318, p-value = 0.092) (Fig 6). In contrast, tree diameter at breast height was larger for female (mean 95 ± 61 cm) than for male bats (mean 71 ± 62 cm) (KS D(20, 39) = 0.414, p-value = 0.016), and was also larger for bats as a whole (mean 78 ± 61 cm) compared to random trees (mean 41 ± 30) (KS D(62) = 1.0, p-value < 0.001) (Fig 7).

A total of nine maternity roost trees were identified in six tree species, *Eucalyptus robusta*, lychee (*Litchi chinensis*), mango (*Mangifera indica*), paperbark *(Melaleuca quinquenervia)*, ‘ōhi‘a (*Metrosideros polymorpha*), and African tulip (*Spathodea campanulata*) (Fig 5, see S3 Appendix), at locations ranging in elevation from 12–894 m above sea level (asl). Height of trees used as maternity roosts ranged from 14 to 26 m (mean 19 ± 4 m; n = 9) (Fig 6) while DBH ranged from 32 to 268 cm (mean 103 ± 73 cm; n = 9) (Fig 7).

We modeled day-roost selection at the tree-level with 91 choice sets for 45 (18 female, 24 male, 3 unknown) unique ‘ōpe‘ape‘a that included the habitat attributes of 55 unique trees, three of which were used by multiple bats (Table 2). The top model for predicting tree roost selection represented 43% of the relative model weight and included predictors related to tree height, tree diameter, canopy cover, and edge (Table 3). Three additional models accounted for another 47% of model weight and included various combinations of the same variables as that of the top model. Models with tree-level variables characterizing the relative prevalence of dominant tree species and native ornon-native status did not contribute any explanatory power (S1 Appendix). Model validation tests indicated that the top-ranked model correctly identified the true roost 74% of the time, and other highly ranked models were assessed from between 68 to 71% (all better than expected due to chance) (Table 3).

**Table 2:** Descriptive statistics of predictor variables used in discrete choice models of tree-level roosts used by ‘ōpe‘ape‘a (*Lasiurus semotus*) (n = 63) and associated random trees (n = 182). Categorical variables are described as the proportion (Prop) of observations for each of used and random tree groups.

	Used trees		Random trees	
	Mean ± SD	Prop (%)	Mean ± SD	Prop (%)
Tree height (m)	19 ± 8		15 ± 10	
Tree diameter at breast height (cm)	78 ± 61		41 ± 30	
Species—*Eucalyptus* spp.		8		8
Species—*Melaleuca quinquenervia*		23		4
Species—*Metrosideros polymorpha*		20		12
Species—Other		49		75
Origin—native		21		15
Origin—nonnative		79		85
Canopy cover—closed (≥60%)		33		44
Canopy cover—open (<60%)		67		56
Edge—present		68		52
Edge—not present		32		48

**Table 3:** Top-ranked tree-level roost selection models for ‘ōpe‘ape‘a (*Lasiurus semotus*). See Table 2 for description of model variables and Table 4 for output of the top-ranked models. Only models with ΔAICc ≤ 4.0 are reported here (see S1 Appendix for full listing of all tree-level models). LogLik = loglikelihood; ΔAICc = difference of AICc between a model and the model with the smallest AICc (Akaike’s information criterion adjusted for small sample size); DF = degrees of freedom; Weight = relative model weight. Performance is the percentage of model validation tests that correctly predicted roost use.

Model	LogLik	ΔAICc	DF	Weight	Performance
Height + DBH + Canopy cover + Edge	-34.5	0.0	4	0.43	74%
Height + DBH + Edge	-36.0	1.0	3	0.27	71%
DBH	-38.8	2.5	1	0.13	68%
Height + DBH + Canopy cover	-37.2	3.4	3	0.08	71%

**Fig 8:**
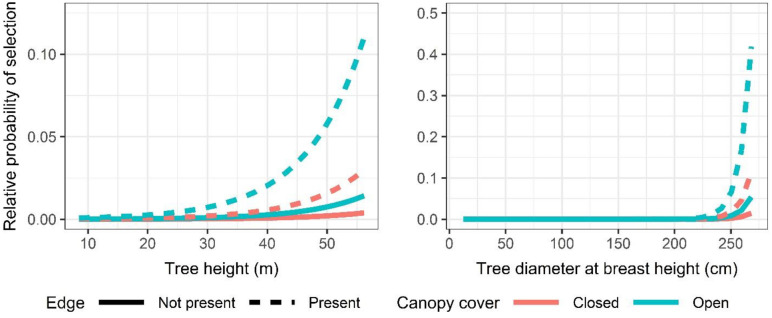
Relative probability of ‘ōpe‘ape‘a (*Lasiurus semotus*) roost selection for the top-ranked tree-level model given the observed range in tree height and tree diameter at breast height, canopy cover (closed and open), and whether roost trees were located adjacent to open areas (edge—present; edge—not present).

The top model for predicting tree roost use indicated that selection was positively related to tree height and tree DBH (Table 4). However, selection of these attributes was only apparent when tree height and DBH was greater than about 20 m and 225 cm, respectively (Fig 8). Selection of tall, large diameter trees was strongest for those with relatively open compared to closed canopies, and for trees that were situated at forest edges.

**Table 4:** Coefficients, standard errors (SE), p-value, and confidence limits [lower—LCL 95%; upper—UCL 95%] of predictor variables in the four top-ranked (ΔAICc ≤ 4.0) tree-level discrete choice models (S1 Appendix) for ‘ōpe‘ape‘a (*Lasiurus semotus*). Reference category for canopy cover was “closed,” and for edge was “not present.” See Methods and Table 2 for parameter descriptions.

Variable	Coefficient	SE	z	p-value	LCL 95%	UCL 95%
Height	0.104	0.037	2.830	0.005	0.032	0.176
DBH	0.012	0.006	2.084	0.037	0.001	0.023
Canopy cover—open	1.304	0.557	2.339	0.019	0.211	2.396
Edge—present	2.048	0.696	2.943	0.003	0.684	3.412
Height	0.110	0.036	3.062	0.002	0.039	0.180
DBH	0.010	0.005	1.960	0.050	0.000	0.021
Canopy cover—open	2.356	0.715	3.294	0.001	0.954	3.757
DBH	0.035	0.008	4.497	<0.001	0.020	0.056
Height	0.109	0.037	2.965	0.003	0.037	0.181
DBH	0.014	0.006	2.349	0.019	0.002	0.025
Edge—present	1.617	0.567	2.850	0.004	0.505	2.729

### Stand-level analyses

A total of 123 roost stands were used by 90 bats (29 female; 58 male; 3 unknown sex). Of these individuals, 23 bats (3 female; 20 male) used multiple stands (range 2–6; mean 3 ± 1). We also observed two roost stands used at different times by multiple bats (range 3–4; mean 4 ± 1) during the study period. The composition of tree species in stands within which roosting bats were located were primarily non-native plantation species (e.g., *Eucalyptus* spp.) and invasive species (e.g., *Falcataria moluccana*), although the native ‘ōhi‘a (*Metrosideros polymorpha)*, was the dominant component for about a quarter of observed day-roost stands (Table 5).

**Table 5:** Descriptive statistics of predictor variables used in discrete choice models of stand-level roosts used by ‘ōpe‘ape‘a (*Lasiurus semotus*) (n = 128) and associated random stands (n = 410). Categorical variables are described as the proportion of observations in used and random stand groups.

	Used stands		Random stands	
	Mean ± SD	Prop (%)	Mean ± SD	Prop (%)
Canopy height (m)	20 ± 7		16 ± 7	
Edge—distance to forest edge (m)	38 ± 67		97 ± 158	
Topographic position	-1 ± 22		0 ± 17	
Slope (degrees)	5 ± 5		6 ± 4	
Species—*Eucalyptus* spp.		31		22
Species—*Falcataria moluccana*		5		12
Species—*Metrosideros polymorpha*		30		33
Species—Other		34		33
Origin—native		22		21
Origin—nonnative		67		63
Origin—mixed		12		16
Canopy cover—closed (>60%)		41		55
Canopy cover—open (25–60%)		27		21
Canopy cover—scattered (<25%)		32		24

**Fig 9:**
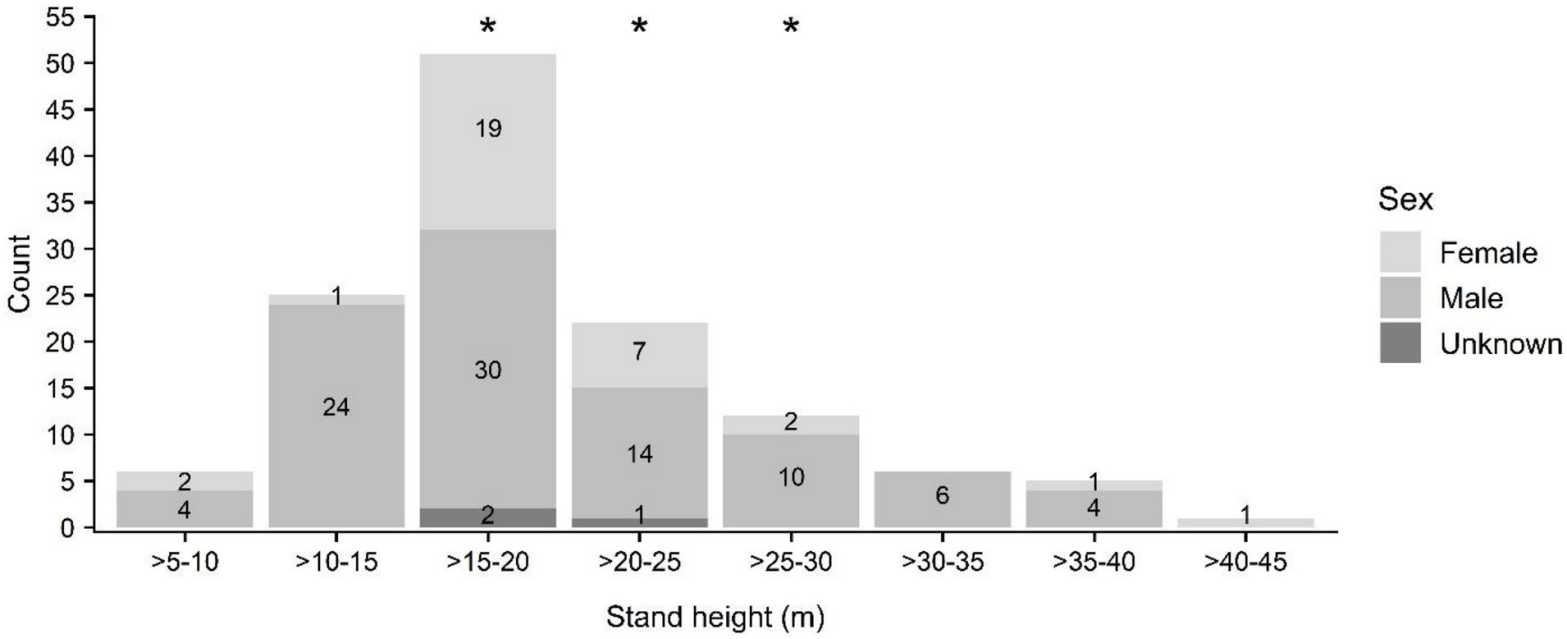
Eight of stands by sex used as roosts by ‘ōpe‘ape‘a (*Lasiurus semotus*). Height classes observed used as maternity roosts are indicated with asterisks.

**Fig 10:**
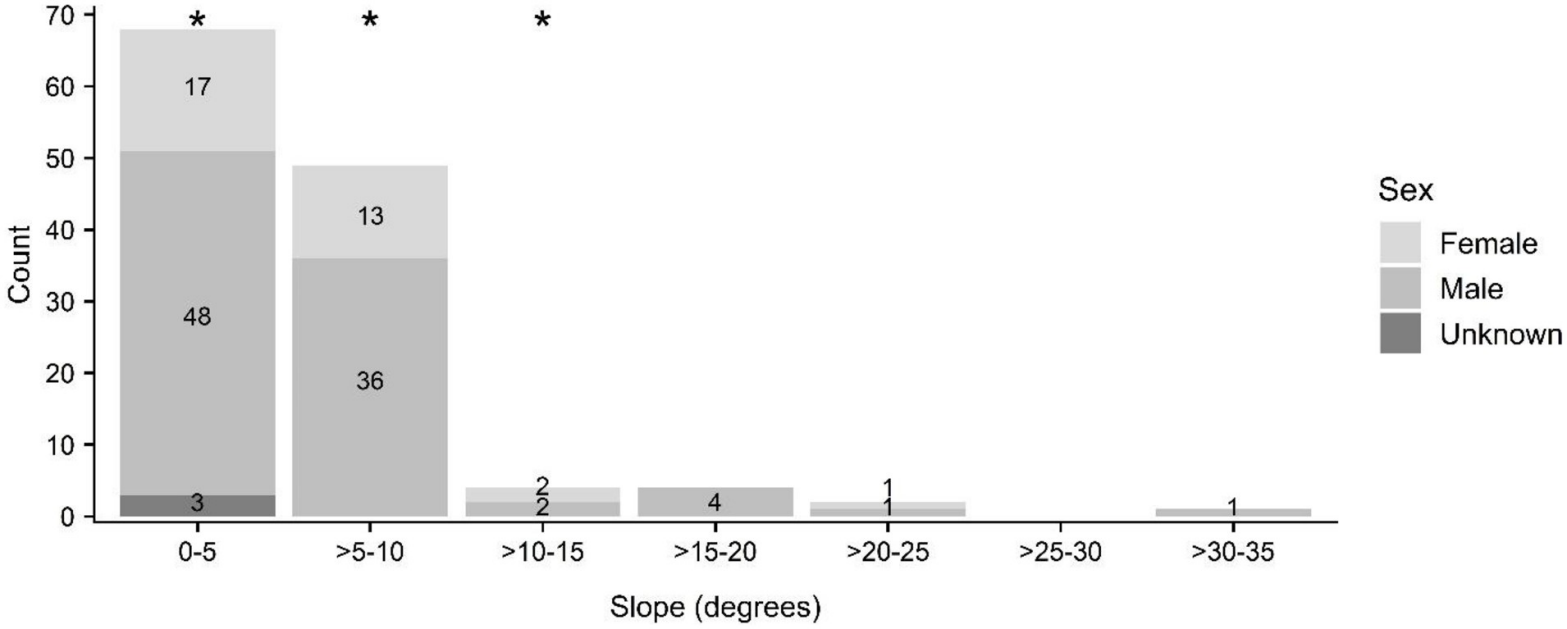
Slope of stands by sex used as roosts by ‘ōpe‘ape‘a (*Lasiurus semotus*). Slope classes observed used as maternity roosts are indicated with asterisks.

The canopy height of stands used by roosting bats (mean 20 ± 7 m) differed significantly from random for bats as a whole (KS D(128) = 1.0, p-value < 0.001) (Fig 9), but not between male and females (female: mean 20 ± 7 m; male: mean 20 ± 7 m; KS D(33,92) = 0.224, p-value = 0.141). Likewise, stands used by roosting bats were on slopes (mean 6 ± 5 m) that differed significantly from random for bats as a whole (KS D(128) = 0.985, p-value < 0.001) (Fig 10), but not between male and females (female: mean 5 ± 4 m; male: mean 6 ± 5 m; KS D(33,92) = 0.072, p-value = 0.999).

We modeled day-roost roost selection at the stand-level with 205 choice sets for 90 unique ‘ōpe‘ape‘a that included the habitat attributes of 123 unique stands, two of which were used by multiple bats. The top model for predicting roost use within a stand included predictors related to stand canopy height, canopy cover, and slope representing 66% of the relative model weight (Table 6). One additional model, accounting for another 10% of model weight, included the same variables as those of the top model as well as topographic position. However, the parameter for topographic position was not significant and indicated that it did not add any explanatory power. Model validation tests indicated that top- and second-ranked models each correctly identified the true roost 48% of the time (both better than expected due to chance) (Table 6).

**Table 6:** Top-ranked stand-level roost selection models for ‘ōpe‘ape‘a (*Lasiurus semotus*). See Table 5 for description of model variables and Table 7 for output of the top-ranked models. Only models with ΔAICc ≤ 4.0 are reported here (see S1 Appendix for full listing of all stand-level models). LogLik = loglikelihood; ΔAICc = difference of AICc between a model and the model with the smallest AICc (Akaike’s information criterion adjusted for small sample size); DF = degrees of freedom; Weight = relative model weight. Performance is the percentage of model validation tests that correctly predicted roost use.

Model	LogLik	ΔAICc	DF	Weight	Performance
Height + Canopy cover + Slope	-84.1	0.0	4	0.66	48%
Height + Canopy cover + Topographic position + Slope	-85.0	3.8	5	0.10	48%

**Fig 11:**
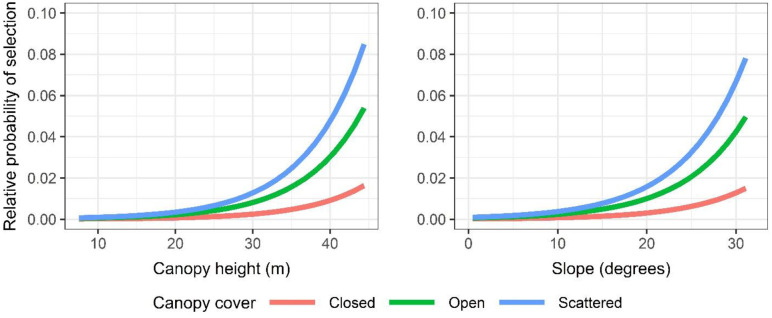
Relative probability of roost selection by ‘ōpe‘ape‘a (*Lasiurus semotus*) predicted by the top-ranked stand-level model given the observed range in canopy height, slope (degrees), and canopy cover (closed, open and scattered).

The top model for predicting roost use at the stand-level indicated that selection was positively related to canopy height and slope (Table 7). However, selection of these attributes was only apparent when canopy height and slope was greater than about 20 m and 10 degrees, respectively (Fig 11). Similar to roost selection at the tree-level, use was strongest for stands composed of scattered and open cover relative to closed canopied settings. Slope likely appeared as a significant variable in the discrete choice model because of steep-slope outliers within choice sets.

**Table 7:** Coefficients, standard errors (SE), p-value, and confidence limits [lower—LCL 95%; upper—UCL 95%] of the top-ranked (ΔAICc ≤ 4.0) stand-level discrete choice models (S1 Appendix) for ‘ōpe‘ape‘a (*Lasiurus semotus*). Reference category for canopy cover is “closed.” See Table 5 for parameter descriptions.

Variable	Coefficient	SE	z	p-value	LCL 95%	UCL 95%
Canopy height	0.132	0.024	5.507	<0.001	0.085	0.178
Canopy cover—open	1.198	0.384	3.115	0.002	0.444	1.951
Canopy cover—scattered	1.651	0.413	3.996	0.000	0.841	2.461
Slope	0.144	0.046	3.167	0.002	0.055	0.233
Canopy height	0.132	0.024	5.491	<0.001	0.085	0.179
Canopy cover—open	1.194	0.386	3.095	0.002	0.438	1.950
Canopy cover—scattered	1.628	0.418	3.889	0.000	0.807	2.448
Topographic position	0.004	0.007	0.504	0.614	-0.010	0.017
Slope	0.151	0.046	3.250	0.001	0.060	0.242

## Discussion

This work represents the most comprehensive study to date of roost selection by ‘ōpe‘ape‘a. Bats roosted in a variety of tree species and in an assortment of habitat stand types, including both native and non-native habitats. Because resource selection is inherently a scale-dependent process [49], we examined roost habitat use at multiple scales—perch, tree, and stand. Our results are largely consistent with the findings of other studies of foliage-roosting, insectivorous bats [24, 50].

At the perch level, ‘ōpe‘ape‘a selected for attributes that may aid in protection from predators [17] and provide microclimate and thermoregulatory benefits [23]. Perch canopy cover was bimodally distributed, with most observations at both extremes of sparse and dense. This may reflect the overall structure of selected trees, which consisted of both low statured, open-canopy trees (e.g., ‘ōhi‘a, *Metrosideros polymorpha*), and relatively large, dense-canopy trees (e.g., lychee, *Litchi chinensis*). Perches selected in relatively sparse perch canopy cover may be explained by increasing the ease of navigation and facilitation of predator detection [16]. Moreover, ‘ōpe‘ape‘a likely evolved with sparse canopy trees such as ‘ōhi‘a (*Metrosideros polymorpha*) [51] from their first arrival on the Hawaiian archipelago approximately 1.3 Ma [6]. Perches with sparse canopy cover were well represented in our data, and their use might also be a function of low roost fidelity common to other foliage-roosting species (e.g., [16, 26, 52]). Data on roost fidelity indicate that transient roosts typically occurred in sparse vegetation that may provide little, other than temporary (<24 hrs) resting places [53]. In contrast, we generally noted consistent occupancy over multiple reproductive seasons, particularly by family groups, at perches with relatively high canopy cover and large, densely foliated trees. Moreover, within these dense and large trees, several bats used more than one perch within the same roost tree and several trees were used by more than one bat concurrently; a behavior only observed in large trees, with dense canopies. This includes one very large, old-growth lychee (*Litchi chinensis*) that had 17 distinct perches over the course of the study [53]. Further investigation of roost structure and fidelity would be useful to better parse out this relationship.

The preference by tree bats for an elevated perch likely facilitates safe access to the roost and may reduce its exposure to a ground-based predator and increase the difficulty for a predator climbing up to the roost [16]. No information exists on the extent of predation risk to roosting ‘ōpe‘ape‘a, but bat odors at roost can attract potential predators [54], and rodents introduced to islands are known to affect tree bat species (e.g., [55]). Few invasive vertebrates are more problematic to island biota than rats, particularly black rats (*Rattus rattus*) because they are so widespread [56]. Given the strong climbing abilities of rats, predation risk may be partly determined by tree physiognomy and bat behavior at roosts. Observations with thermal video during our study of ‘ōpe‘ape‘a at roost revealed considerable nocturnal rat activity, but one that appears restricted to trees of low stature (e.g., ≤12 m; [57]) or with a lateral, bifurcated branching structure, and absent from tall, vertically oriented single-stemmed trees [53]. Notably, ‘ōpe‘ape‘a frequently perch on the extremities of branchlets and leaf petioles, which may allow for rapid departure from a roost in the presence of an arboreal predator.

Observations of roost perches in the lower halves of trees were uncommon (13 of 69 perches [<20%]) and those were generally located in relatively taller trees. A notable exception included a perch situated at a very low height (1.8 m above ground) within an uluhe (*Dicranopteris linearis*) matted fern thicket not previously observed as roosting habitat. It is possible that ‘ōpe‘ape‘a use such dense vegetation when seeking a thermoregulatory benefit (e.g., comparable to roosting in forest litter by eastern red bats (*Lasiurus borealis*; [58, 59]). Although torpor has not been documented in ‘ōpe‘ape‘a, it has been speculated that they may engage in short bouts of torpor during inclement weather events to help preserve energy stores [7, 15, 60]. We observed the bat at perch in the uluhe (*Dicranopteris linearis*) thicket during the day and noted that the bat appeared to be in a low-energy state and did not flush or attempt to flush when inadvertently disturbed during the course of tracking and honing its radio signal. The habitat area was one of the highest elevation roosts recorded (1,661 m asl) where mean low and high temperatures when the bat was captured and tracked to roost in February 2020, were 5.6 and 14.4 degrees Celsius [61]. Although we were unable to measure bat skin temperature or roost temperature, the observed behavior may indicate use of torpor and selection of thermally insulating roosts by ‘ōpe‘ape‘a under certain conditions. Further investigation would be useful to confirm and evaluate torpor by ‘ōpe‘ape‘a, as it is possible that local weather conditions may have contributed to the bat using this roost for its thermal benefits.

Perch aspect was significantly oriented toward the southwest, which may indicate bats selected roost perches that minimized exposure to inclement weather associated with the prevailing northeasterly “tradewinds” in Hawai‘i [62]. Such behavior has also been noted in other North American species of *Lasiurus*. For example, Klug *et al*. [63] demonstrated a preference for roosting on the lee-side of trees by both resident and non-resident migrating hoary bats (*Lasiurus cinereus*). Increased radiant heating and protection from prevailing winds at southeast-facing roosts used by lactating *Lasiurus cinereus* in North America reduced energy expenditure from heat loss [23]. Sunlight exposure may also be a factor in the preference by ‘ōpe‘ape‘a for a southerly perch aspect where exposure to sunlight in the evenings may facilitate rewarming before roost emergence and nightly foraging. Perch aspect was not found to differ between reproductive (May–Sept) and non-reproductive (Oct–Apr) seasons. This is likely due to the low latitude of Hawai‘i resulting in a relatively uniform day-length and sun that result in solar radiation patterns exhibiting little seasonal variation in solar radiation. Moreover, perch aspect did not differ significantly between sexes and between maternity versus non-maternity roosts, indicating that individuals may be able to accommodate sex- and reproduction-specific thermoregulation needs, given Hawai‘i’s relatively moderate climate, by selecting perch locations in relation to aspect and other factors influencing exposure.

We recorded bats roosting in 19 different tree species, inclusive of species used in non-native timber plantations, fruit and ornamental trees in urban settings, as well as in native species. The most used species, paperbark (*Melaleuca quinquenervia*), is almost exclusively used as a windbreak and/or to demarcate agricultural grids or boundaries on Hawai‘i Island, and was consistently found as part of an orchard windrow or edge when used by roosting ‘ōpe‘ape‘a. Because the proximity to forest edges and proximity to roads made the radio signals of tagged bats easier to detect compared to interior forests, the prevalence of paperbark (*Melaleuca quinquenervia*) as roost trees in these data may represent sampling bias. Notably, we did not find bats roosting in the orchard trees themselves (i.e., *Macadamia* spp.). Bats consistently selected roosts in windrows surrounding orchards, indicating that these windrows provided valuable habitat for ‘ōpe‘ape‘a in terms of both shelter and proximity to food resources. We surmise that ‘ōpe‘ape‘a may play a role in suppressing insect pests in agricultural areas sheltered by paperbark (*Melaleuca quinquenervia*) windrows. The endemic species ‘ōhi‘a (*Metrosideros polymorpha*) was the second-most used species represented in our data, which may be a factor of it being widespread and major forest component throughout Hawai‘i Island. In contrast, the endemic koa (*Acacia koa*) was not recorded as a bat roost at any time during our study despite capture and tracking efforts within koa-dominated and mixed koa forests, as well as documented foraging activity [14, 64] within these relatively common land-cover types. The notable absence of observed bat roosts in koa (*Acacia koa*) may be attributable to the species’ leaf structure, which is composed of vertically oriented phyllodes that function to reduce light interception and plant heat loading [65] but may in turn lessen potential cover for bats. However, although bats may avoid certain tree attributes and species, the wide diversity of trees used as roosts by ‘ōpe‘ape‘a during this study indicate that they are flexible in their choice of tree species–a finding consistent with observations for other species of *Lasiurus*. For example, Andersen and Geluso [26] found no apparent selection for tree species by western red bats (*Lasiurus blossevilli*), and instead suggest that roost selection was driven by foliage density. Likewise, Perry and Thill [66] recorded coniferous and deciduous roost tree use by *Lasiurus cinereus* at a ratio equivalent to their proportional representation in the landscape.

Although the limited sample size of maternity roosts in our study precluded comparative analyses, the smaller subset of tree species used by reproductive females may indicate a preference, albeit non-exclusive, for particular trees. Maternity roosts were identified in six tree species, including plantation species such as swamp mahogany (*Eucalyptus robusta*) and paperbark (*Melaleuca quinquenervia*), introduced fruit trees lychee (*Litchi chinensis*) and mango (*Mangifera indica*), the invasive species African tulip (*Spathodea campanulata*), and the native endemic ‘ōhi‘a (*Metrosideros polymorpha*). Notably, we located roost “hot-spots” in large and densely foliated lychee (*Litchi chinensis*) and mango (*Mangifera indica*) trees that were consistently used by both multiple maternity groups and single bats in different parts of the same tree and over several reproductive seasons. Maternity roosts in both paperbark (*Melaleuca quinquenervia*) and lychee (*Litchi chinensis*) were used as maternity roosts over several maternity seasons and maternity roosts in lychee (*Litchi chinensis*) and mango (*Mangifera indica*) were sometimes found to be in use concurrently with other single bats roosting in other parts of the same tree. Because some individuals were not marked and identified among years, it is unclear if these “hot-spots” were due to natal philopatry or were simply favored by otherwise unrelated bats. Targeted multi-year capture or genetic studies could improve understanding of these roost clusters.

‘Ōpe‘ape‘a selected roost trees with physical features that differed from randomly available trees. Roost trees were larger in both height and diameter than randomly sampled trees. These results differ from those observed for reproductive female *Lasiurus cinereus* (64, 23), which did not significantly select roosts with those attributes in the areas studied in the northern part of that species’ range. However, our observations are in agreement with those made in other studies of tree-roosting bats in the genus *Lasiurus* (e.g., *Lasiurus borealis* and *Lasiurus seminolus* [22, 67–69]). Larger trees may provide easier access to and from the roost perch and be more readily relocated than closed canopied trees in contiguous forest stands [16]. However, roost preference by ‘ōpe‘ape‘a was only apparent for tree heights and diameters greater than 20 m and 225 cm, respectively. Whereas about a quarter of surveyed roost trees were taller than 20 m, only one roost tree was greater than 225 cm DBH—a mature lychee (*Litchi chinensis*) located in an open setting within an urban, residential area. The landowners where this tree was located reported consistent bat use over several decades and the tree accounted for 17 distinct roost perches throughout our study period. We observed its use as a maternity roost, and concurrently by other bats, over four maternity seasons (2018–2021). As such, it accounted for the most choice sets (n = 7) of any occupied roost tree in our discrete choice models. Its consistent occupancy over several years and among numerous individual bats was a major factor influencing large-diameter trees being selected by our model as important for predicting roost use by ‘ōpe‘ape‘a. Tree canopy cover was generally more open at roosts used by ‘ōpe‘ape‘a than at randomly available trees, a result similar to observations of *Lasiurus cinereus* [23] and other tree-roosting bats in North America [16]. Selection of relatively open canopied trees also extended to the use of roosts situated at the edge of open areas and within stands composed of dispersed trees, a preference likewise apparent in *Lasiurus cinereus* [63] and *Lasiurus blossevilli* [26]. As with the preference for relatively tall or emergent trees, selection for open structured trees and stands may ease roost access by providing gaps or flyways [70], or simply provide nearby forest-edge habitat favored by foraging insectivorous bats such as *Lasiurus cinereus* [71].

Bats roosted in a variety of forest stands including urban, agricultural, tree plantation, mixed native, and non-native habitats. Roost selection at the forest-stand level indicated that selection was positively related to canopy height, canopy cover, and slope but was only apparent when canopy height and slope were greater than 20 m and 15 degrees, respectively. Selection was also greatest for stands composed of scattered and open cover relative to closed canopied habitats. Selection for canopy height was most apparent in both open and scattered habitats. Vonhoff and Barclay [16] found that tree-roosting bats in their study preferred tall trees surrounded by relatively open canopy. These conditions may provide easier access to and from the roost [16] as well as additional thermal benefits [72]. Willis and Brigham [23] also found that reproductive female *Lasiurus cinereus* in Saskatchewan, Canada, selected roost trees in forest patches with reduced density, particularly on the southeast sides of roost trees. Benefits of such roosting habits could be two-fold—reduced forest density could increase sun exposure and radiant heat at the roost, as well as serve as flyways for approaching and departing roosts among foliage.

Given the topographic variation of our study area (see [73]), most roost stands were situated on landscapes with some amount of slope. On average most sites were not exceptionally steep (≈6 degrees), but at some locations slope could range up to 32 degrees, particularly in terrain deeply incised with gulches. The potential benefit of a steep slope may include the increased height and the contrast it provides a roost, relative to surrounding vegetation, and the attendant ease of roost access. However, contrary to findings for other bat species (e.g., *Lasiurus borealis* [52]), we did not find a significant effect of topographic position (i.e., drainage bottom versus adjacent slope versus ridge-top) on roost selection by ‘ōpe‘ape‘a. This result may have been due to the low prevalence of deep gulches in our study area. On the other hand, roosts on steep slopes may have been under-represented because of the difficulty of detecting telemetry signals originating within gulches and not in “line-of-sight” with observers tracking bats; such an effect was thought to have limited tracking of ‘ōpe‘ape‘a in a study on the island of Maui [20].

Discrete choice models were an effective method to identify day-roost preferences of ‘ōpe‘ape‘a. However, several factors may have affected our ability to elucidate further habitat use patterns. We were able to track as many as 70% of the 130 radio-tagged bats to a roost stand, but only about half that many to a roost-tree and perch. Given the broad elevation span and wide range of land-cover types surveyed, a larger sample size may be required to determine whether stronger roost preferences are evident than those revealed in our study. Further, sampling focused on a specific landcover class or spatial areas might reveal localized preferences. Specialization within population groups (e.g., males versus female bats during the reproductive period) may also obscure patterns when examined in aggregate, particularly if individual variation in resource use within groups is large. Seasonal population-level movement and segregation (e.g., males migrating out of the lowlands and into the uplands during the post-lactation period; [60]) may also affect bat capture and roost identification. Finally, the extensive trail and road network in lowland settings (< 1,000 m asl) facilitated tracking bats in habitats consisting largely of agricultural and urban/suburban landscapes. This potential sampling bias might have contributed to under-sampling bats in the native forest more prevalent at higher at elevations on Hawai‘i Island and biased modeled roost-use patterns toward individuals tracked for longer periods.

‘Ōpe‘ape‘a used day-roosts in a wide range of tree species of diverse physiognomy, and in both native and non-native habitats. Bats generally chose roost trees larger in diameter, taller in height, lower in canopy cover, and closer to forest edge than randomly available options. This plasticity in roost selection may allow for the species’ broad distribution and capacity to use highly fragmented habitats characteristic of Hawaiian landscapes [7, 15]. However, ‘ōpe‘ape‘a population size and abundance trends are not known [10], and genetically distinct groups with limited gene flow [74] may make populations vulnerable to extirpation. Although this study marks the first directed research for quantifying roost habitat for ‘ōpe‘ape‘a, additional studies tracking females to maternity roosts and evaluating sex-specific and seasonal roost requirements could help inform management efforts aimed at improving maternity and breeding season habitats (e.g. [75]). Additionally, although maternity roosts were found across a range of forest types, tree species and characteristics, evidence indicating bat occupancy of legacy trees and maternal roost clustering, can be used to identify areas of high conservation value. Moreover, obtaining additional roost data in upland habitats may give a better picture of seasonal roosting variation, and sampling focused on specific landcover classes or spatial areas could reveal localized preferences.

In Hawai‘i and elsewhere, habitat restoration and conservation area management have focused on native species out-planting (e.g., [76]). The broad range of tree species used as roosts by ‘ōpe‘ape‘a might allow for flexibility in tree species used for restoring roost habitats, including native species like ‘ōhi‘a (*Metrosideros polymorpha*) and lama (*Diospyros sandwicensis*). Although we did not find bats roosting in koa (*Acacia koa*), this native tree may provide suitable habitat for potential prey items [64, 77]. As such, conservation strategies aimed at protecting or restoring habitat for the benefit of the species could be improved with considerations of both seasonal roosting and foraging requirements [78]. Protection of very large canopy trees identified as legacy maternity sites could provide important habitat for rearing young. Overall preservation of larger diameter and taller trees near edges may support bat habitat needs indicated by preferences revealed by this study. The new information on ‘ōpe‘ape‘a roosting ecology can be used to inform mitigation, protection, and restoration actions aimed at assisting the recovery of the species.

## Supporting information

S1 Appendix Discrete choice model ranking for each of two analyses: (1) tree-level models; (2) stand-level models. See Tables 2 and 5 for description of model variables and Tables 4 and 7 for output of the top-ranked models (ΔAICc ≤ 4.0). LogLik = loglikelihood; ΔAICc = difference of AICc between a model and the model with the smallest AICc (Akaike’s information criterion adjusted for small sample size);); DF = degrees of freedom; Weight = relative model weight. Model variables are abbreviated as follows: height (ht); diameter at breast height (dbh); canopy cover (cc); distance from forest edge (edge); tree species (spp); native versus non-native origin (native); slope (slope); topographic position index (tpi). Both tree-level and stand-level model sets include a null model.
(ZIP)

S2 Appendix Images of ‘ōpe‘ape‘a (*Lasiurus semotus*) at roost perches (left) and the location within the tree marked with red circle (right) in a selection of tree species, (A, B) adult male in rainbow shower (*Cassia x nealiae*), (C, D) adult male in uluhe or false staghorn fern (*Dicranopteris linearis*), (E, F) adult male in lama (*Diospyros sandwicensis*), (G, H) juvenile female in blue gum (*Eucalyptus saligna*), (I, J) adult male in lychee (*Litchi chinensis*), and (K, L) adult female in mango (*Mangifera indica*).
(ZIP)

S3 Appendix Images of adult female ‘ōpe‘ape‘a (*Lasiurus semotus*) with two pups at maternity roost perches in a selection of tree species, (A) swamp mahogany (*Eucalyptus robusta*), (B) lychee (*Litchi chinensis*), (C) mango (*Mangifera indica*), (D) paperbark (*Melaleuca quinquenervia*), (E) ‘ōhi‘a (*Metrosideros polymorpha*), and (F) African tulip (*Spathodea campanulata*).
(TIF)
