# Supplementary figures and images for: Multi-scale assessment of roost selection by ‘ōpe‘ape‘a, the Hawaiian hoary bat (Lasiurus semotus)
Source: PLoS One. 2023 Aug 24;18(8):e0288280. doi: 10.1371/journal.pone.0288280 (PMC10449229; doi:10.1371/journal.pone.0288280)

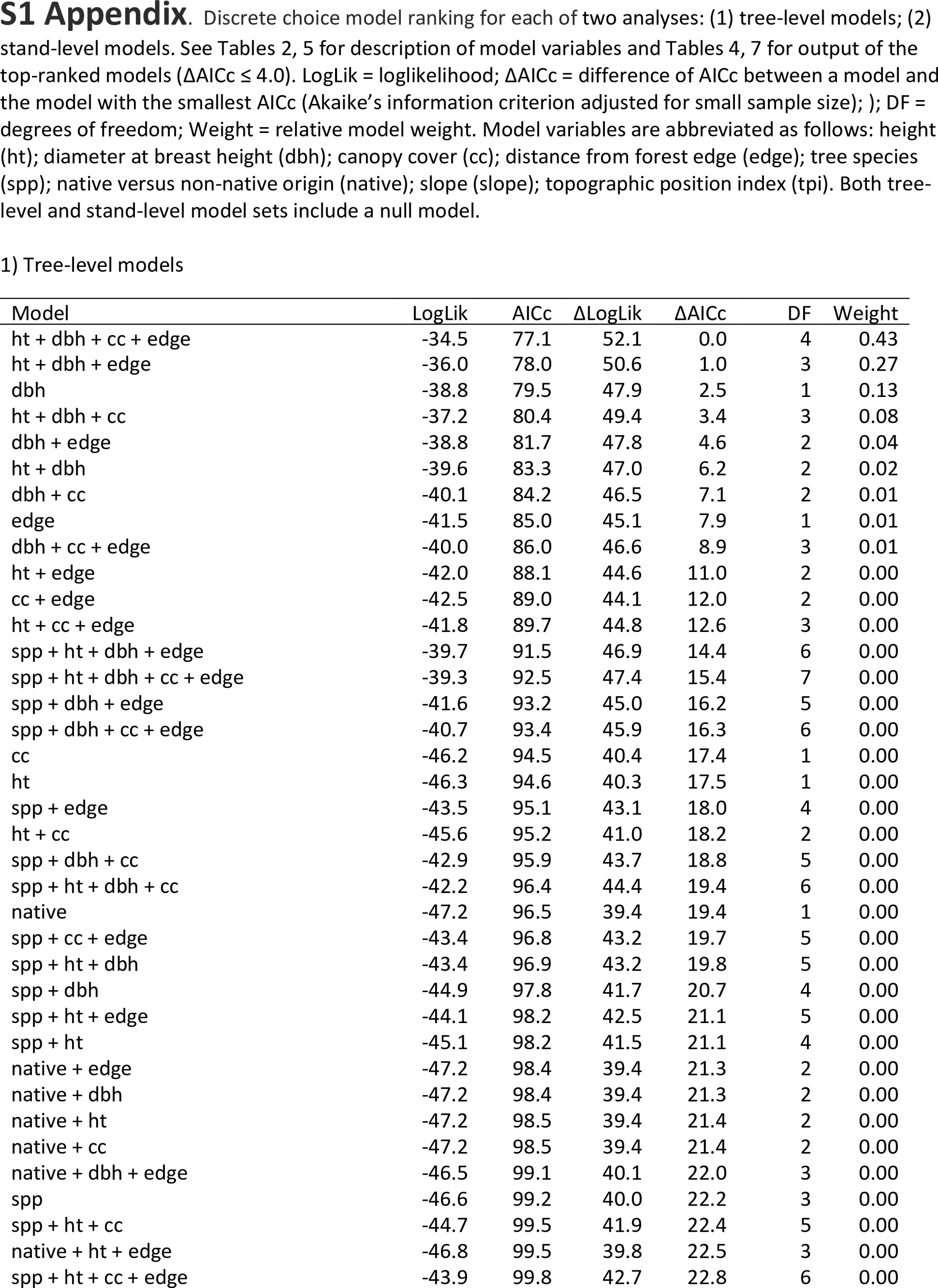

Supplement: S1 Appendix — See Tables 2 and 5 for description of model variables and Tables 4 and 7 for output of the top-ranked models (ΔAICc ≤ 4.0). LogLik = loglikelihood; ΔAICc = difference of AICc between a model and the model with the smallest AICc (Akaike’s information criterion adjusted for small sample size);); DF = degrees of freedom; Weight = relative model weight. Model variables are abbreviated as follows: height (ht); diameter at breast height (dbh); canopy cover (cc); distance from forest edge (edge); tree species (spp); native versus non-native origin (native); slope (slope); topographic position index (tpi). Both tree-level and stand-level model sets include a null model. (ZIP) [file pone.0288280.s001.zip › S1 Appendix p1.tif]

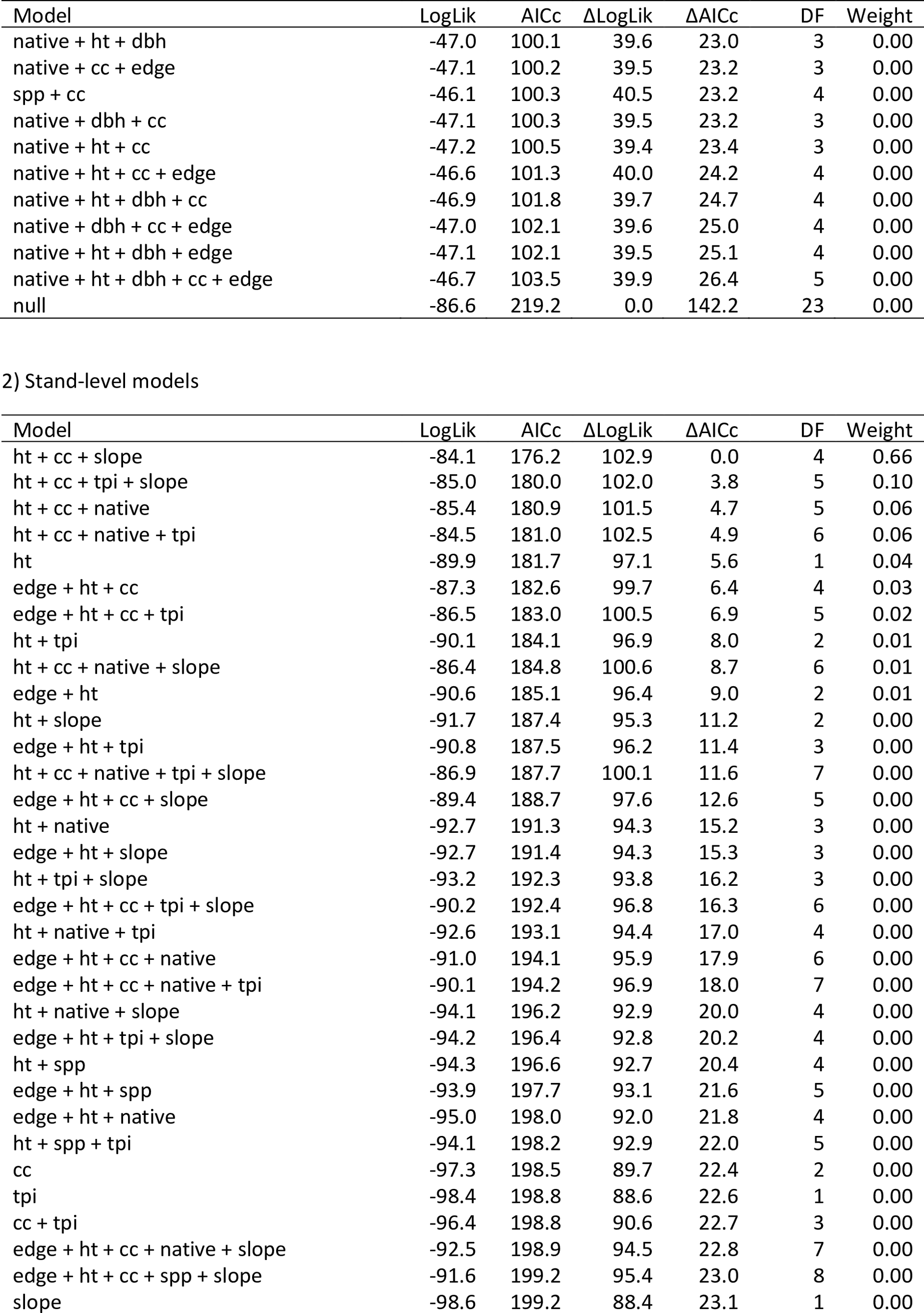

Supplement: S1 Appendix — See Tables 2 and 5 for description of model variables and Tables 4 and 7 for output of the top-ranked models (ΔAICc ≤ 4.0). LogLik = loglikelihood; ΔAICc = difference of AICc between a model and the model with the smallest AICc (Akaike’s information criterion adjusted for small sample size);); DF = degrees of freedom; Weight = relative model weight. Model variables are abbreviated as follows: height (ht); diameter at breast height (dbh); canopy cover (cc); distance from forest edge (edge); tree species (spp); native versus non-native origin (native); slope (slope); topographic position index (tpi). Both tree-level and stand-level model sets include a null model. (ZIP) [file pone.0288280.s001.zip › S1 Appendix p2.tif]

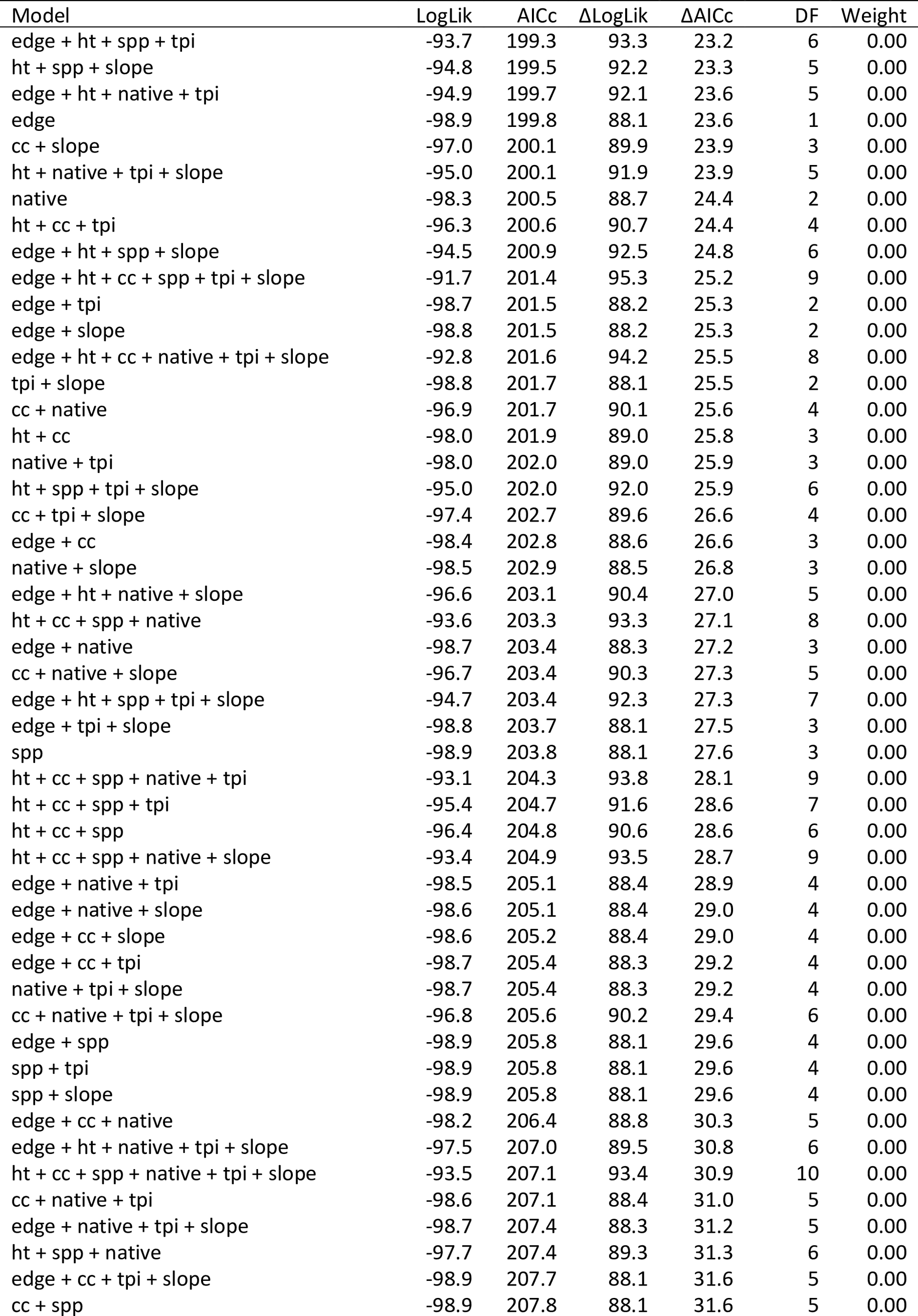

Supplement: S1 Appendix — See Tables 2 and 5 for description of model variables and Tables 4 and 7 for output of the top-ranked models (ΔAICc ≤ 4.0). LogLik = loglikelihood; ΔAICc = difference of AICc between a model and the model with the smallest AICc (Akaike’s information criterion adjusted for small sample size);); DF = degrees of freedom; Weight = relative model weight. Model variables are abbreviated as follows: height (ht); diameter at breast height (dbh); canopy cover (cc); distance from forest edge (edge); tree species (spp); native versus non-native origin (native); slope (slope); topographic position index (tpi). Both tree-level and stand-level model sets include a null model. (ZIP) [file pone.0288280.s001.zip › S1 Appendix p3.tif]

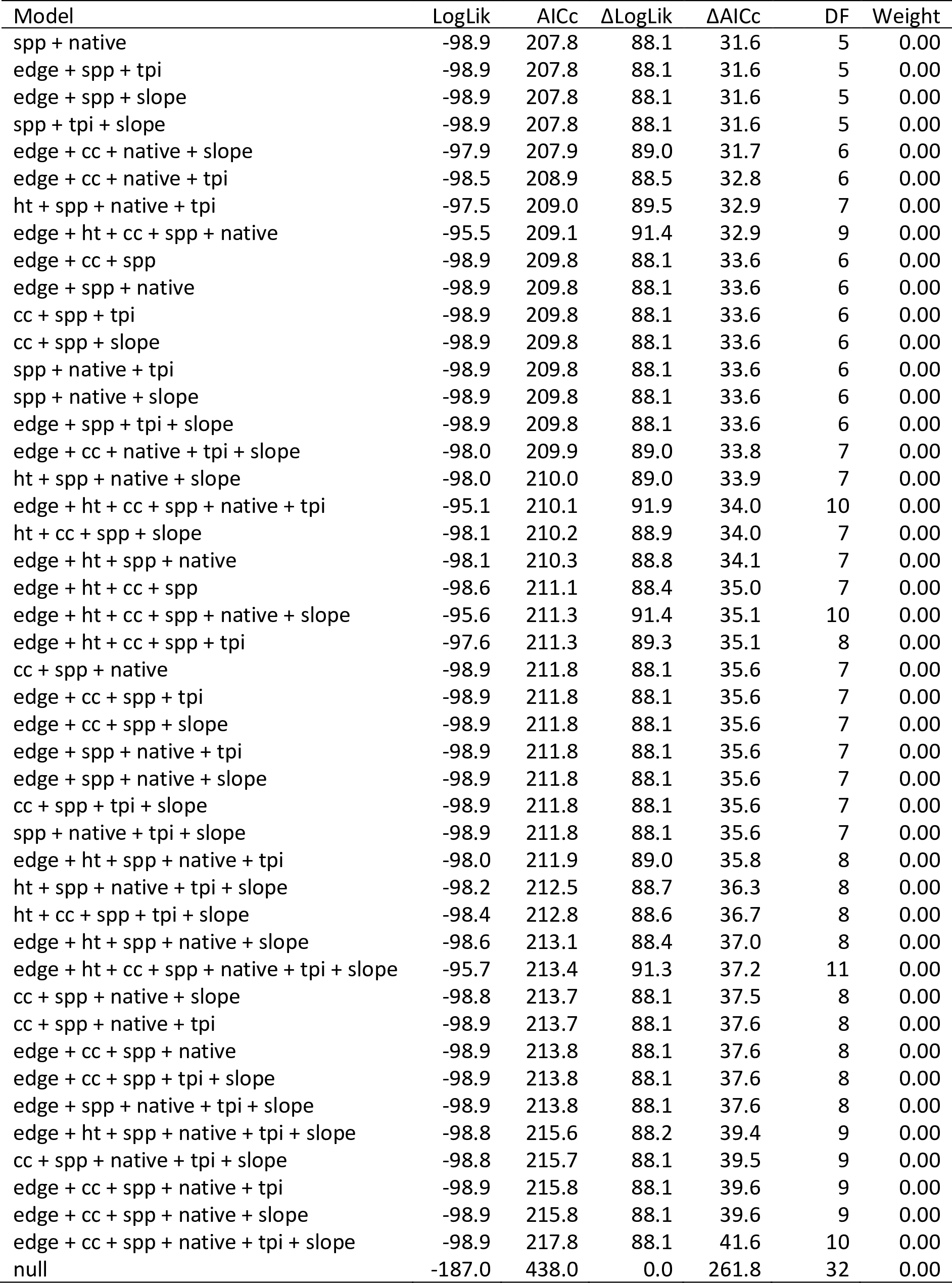

Supplement: S1 Appendix — See Tables 2 and 5 for description of model variables and Tables 4 and 7 for output of the top-ranked models (ΔAICc ≤ 4.0). LogLik = loglikelihood; ΔAICc = difference of AICc between a model and the model with the smallest AICc (Akaike’s information criterion adjusted for small sample size);); DF = degrees of freedom; Weight = relative model weight. Model variables are abbreviated as follows: height (ht); diameter at breast height (dbh); canopy cover (cc); distance from forest edge (edge); tree species (spp); native versus non-native origin (native); slope (slope); topographic position index (tpi). Both tree-level and stand-level model sets include a null model. (ZIP) [file pone.0288280.s001.zip › S1 Appendix p4.tif]

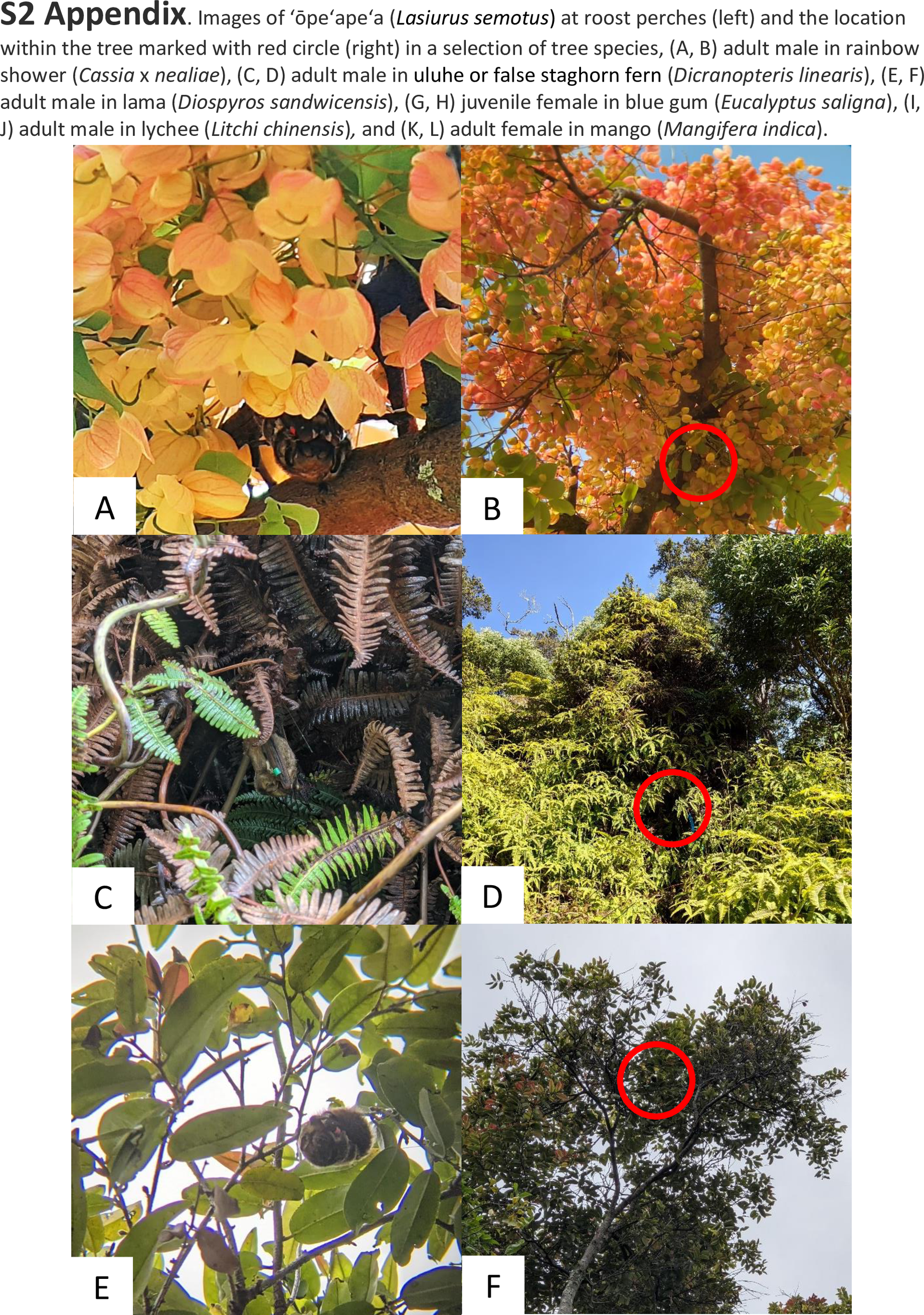

Supplement: S2 Appendix — Images of ‘ōpe‘ape‘a (Lasiurus semotus) at roost perches (left) and the location within the tree marked with red circle (right) in a selection of tree species, (A, B) adult male in rainbow shower (Cassia x nealiae), (C, D) adult male in uluhe or false staghorn fern (Dicranopteris linearis), (E, F) adult male in lama (Diospyros sandwicensis), (G, H) juvenile female in blue gum (Eucalyptus saligna), (I, J) adult male in lychee (Litchi chinensis), and (K, L) adult female in mango (Mangifera indica). (ZIP) [file pone.0288280.s002.zip › S2 Appendix p1.tif]

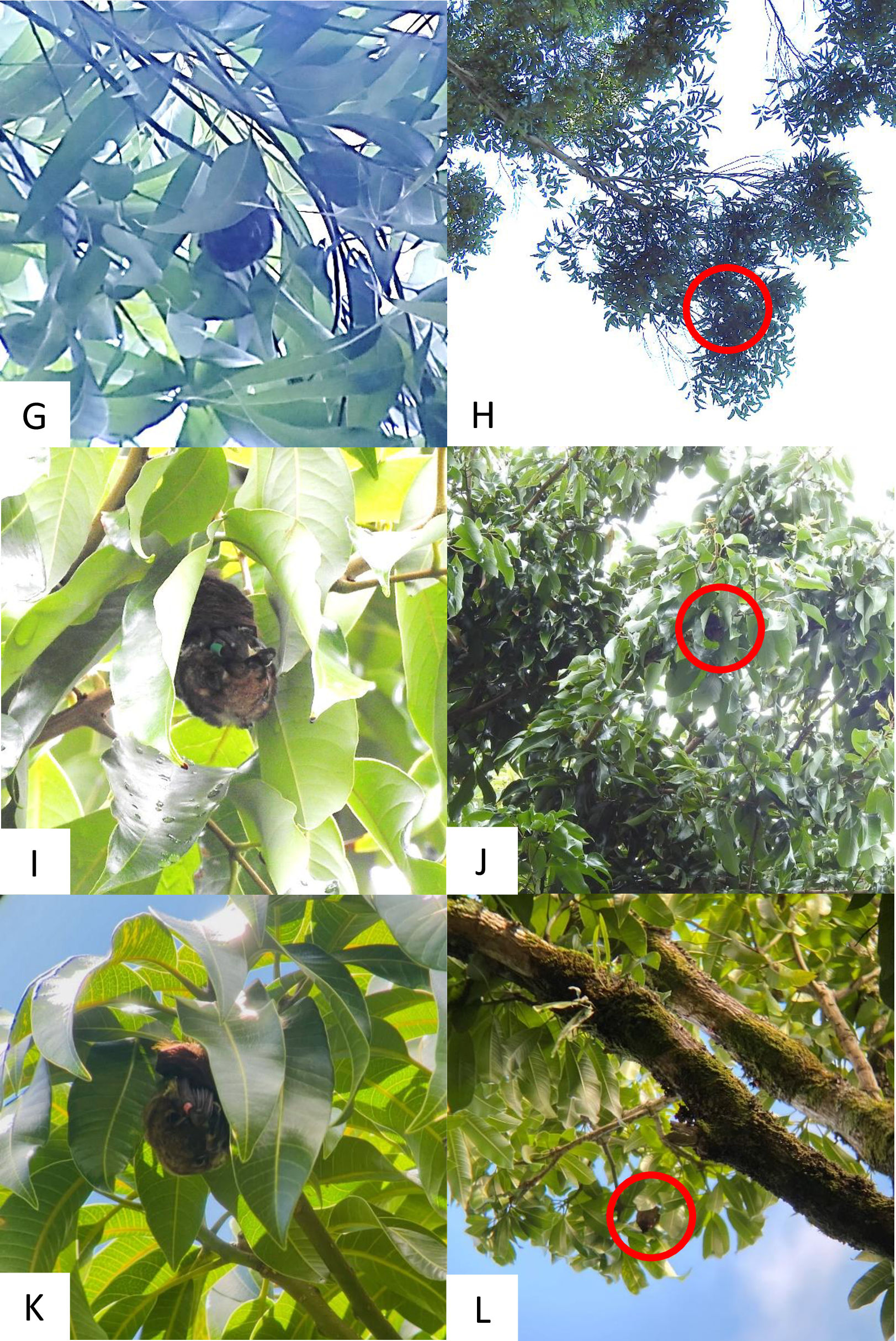

Supplement: S2 Appendix — Images of ‘ōpe‘ape‘a (Lasiurus semotus) at roost perches (left) and the location within the tree marked with red circle (right) in a selection of tree species, (A, B) adult male in rainbow shower (Cassia x nealiae), (C, D) adult male in uluhe or false staghorn fern (Dicranopteris linearis), (E, F) adult male in lama (Diospyros sandwicensis), (G, H) juvenile female in blue gum (Eucalyptus saligna), (I, J) adult male in lychee (Litchi chinensis), and (K, L) adult female in mango (Mangifera indica). (ZIP) [file pone.0288280.s002.zip › S2 Appendix p2.tif]

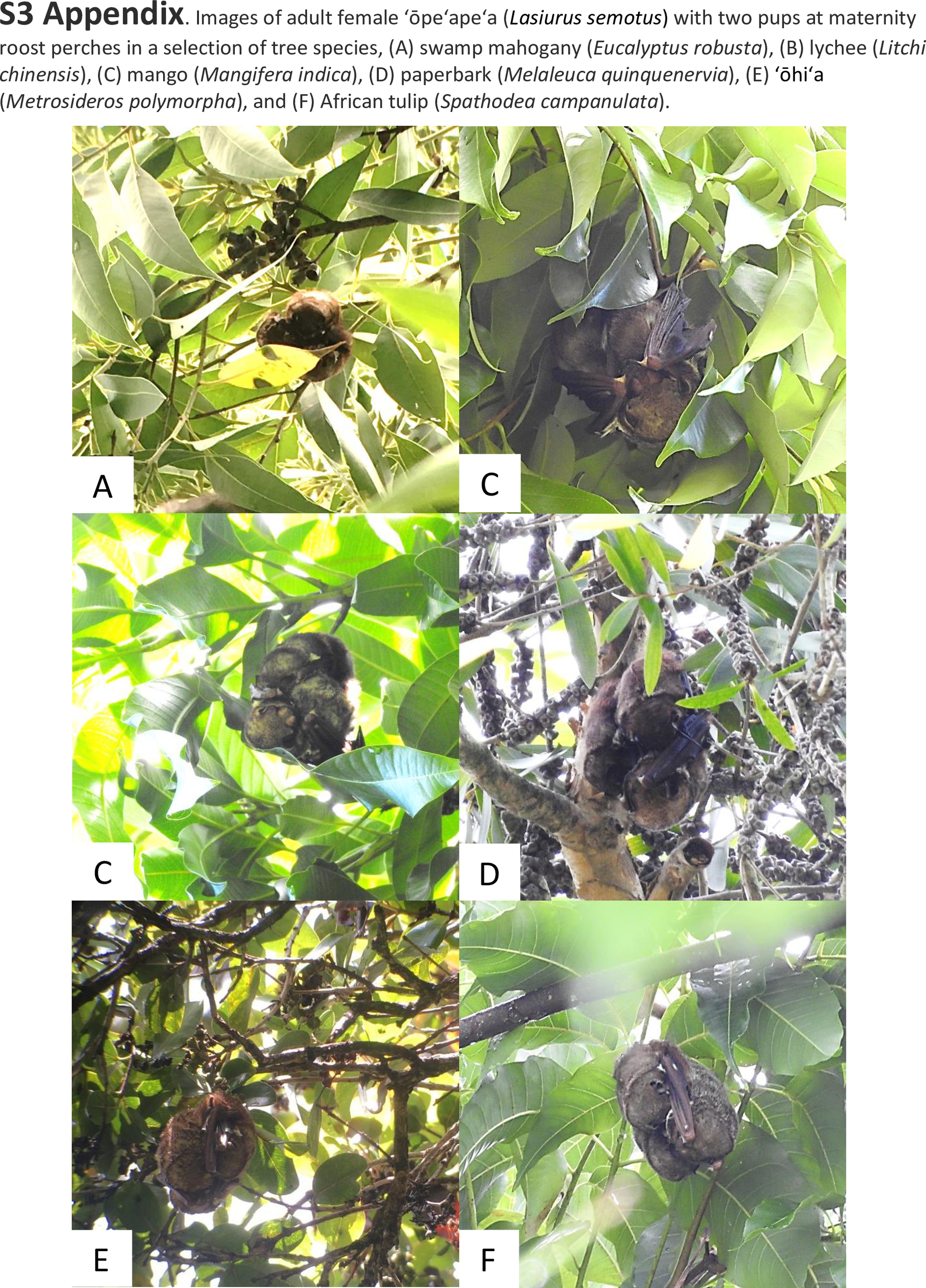

Supplement: S3 Appendix — Images of adult female ‘ōpe‘ape‘a (Lasiurus semotus) with two pups at maternity roost perches in a selection of tree species, (A) swamp mahogany (Eucalyptus robusta), (B) lychee (Litchi chinensis), (C) mango (Mangifera indica), (D) paperbark (Melaleuca quinquenervia), (E) ‘ōhi‘a (Metrosideros polymorpha), and (F) African tulip (Spathodea campanulata). (TIF) [file pone.0288280.s003.tif]
